# Investigation of ‘Head-to-Tail’-Connected Oligoaryl *N*,*O*-Ligands as Recognition Motifs for Cancer-Relevant G-Quadruplexes

**DOI:** 10.3390/molecules22122160

**Published:** 2017-12-06

**Authors:** Natalia Rizeq, Savvas N. Georgiades

**Affiliations:** Department of Chemistry, University of Cyprus, 1 Panepistimiou Avenue, Aglandjia 2109, Nicosia, Cyprus; nataliarizeq@hotmail.com

**Keywords:** pyridyl-oxazoles, *N*,*O*-oligoaryl ligands, G-quadruplexes, conformational transition, C–H activation

## Abstract

Oligomeric compounds, constituted of consecutive *N*,*O*-heteroaromatic rings, introduce useful and tunable properties as alternative ligands for biomolecular recognition. In this study, we have explored a synthetic scheme relying on Van Leusen oxazole formation, in conjunction with C–H activation of the formed oxazoles and their subsequent C–C cross-coupling to 2-bromopyridines in order to assemble a library of variable-length, ‘head-to-tail’-connected, pyridyl-oxazole ligands. Through investigation of the interaction of the three longer ligands (5-mer, 6-mer, 7-mer) with cancer-relevant G-quadruplex structures (human telomeric/22AG and c-Myc oncogene promoter/Myc2345-Pu22), the asymmetric pyridyl-oxazole motif has been demonstrated to be a prominent recognition element for G-quadruplexes. Fluorescence titrations reveal excellent binding affinities of the 7-mer and 6-mer for a Na^+^-induced antiparallel 22AG G-quadruplex (K_D_ = 0.6 × 10^−7^ M^−1^ and 0.8 × 10^−7^ M^−1^, respectively), and satisfactory (albeit lower) affinities for the 22AG/K^+^ and Myc2345-Pu22/K^+^ G-quadruplexes. All ligands tested exhibit substantial selectivity for G-quadruplex versus duplex (ds26) DNA, as evidenced by competitive Förster resonance energy transfer (FRET) melting assays. Additionally, the 7-mer and 6-mer are capable of promoting a sharp morphology transition of 22AG/K^+^ G-quadruplex.

## 1. Introduction

Since the discovery of G-quadruplexes (tetra-stranded nucleic acid helices) in sequences from the human telomere [1,2,3] and from promoters of well-characterized oncogenes [4,5,6], among various other sites in the human genome [7,8], it has become apparent that formation of G-quadruplexes at the expense of more prevalent nucleic acid secondary structures may in fact lead to regulatory effects in various biological processes, such as chromatin remodelling, gene replication and transcription, genomic instability and repair, and apoptosis or survival [9,10,11,12,13]. The ‘rules’ that govern the folding and dynamic interconversions of G-quadruplexes in living systems are not yet fully elucidated, thus new information on their interaction with other molecules may shed light into their dynamic behavior. G-quadruplexes are now recognized as alternative and compelling targets for the development of anticancer drugs [14,15], especially in cases where the DNA-encoded protein is considered ‘nondruggable’, like the product of the c-Myc oncogene [16]. Moreover, interest has arisen in developing chemical ‘tools’ that offer the potential, by means of direct interaction accompanied by a detectable change of their properties, to probe unknown dynamic morphologies and functions of G-quadruplexes in living systems [17,18].

**Figure 1:**
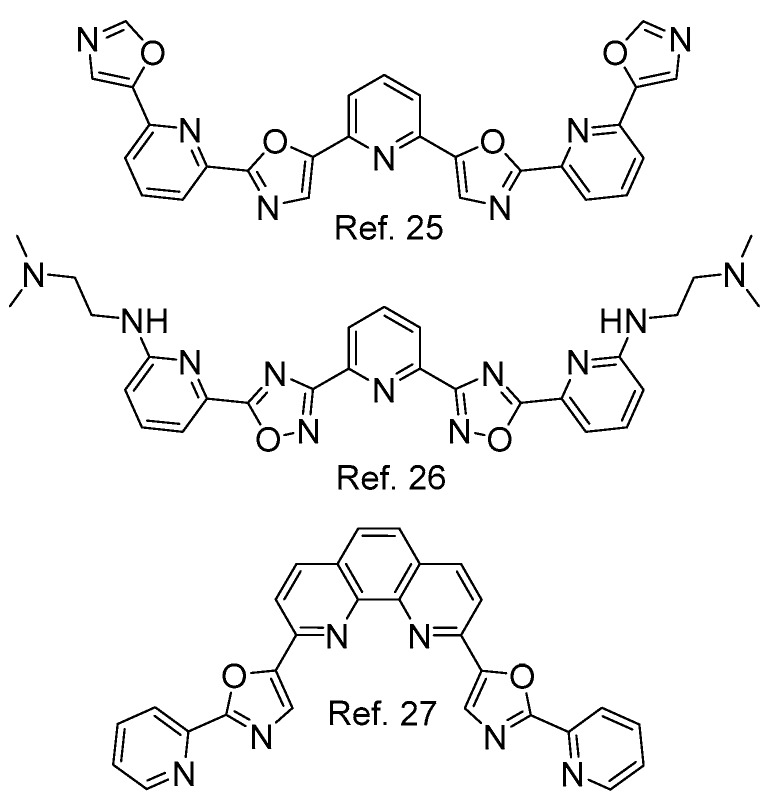
Representative examples of symmetric pyridine/oxazole-derived *N*,*O*-ligands for recognition of G-quadruplexes.

Numerous efforts to develop potent and target-selective G-quadruplex-interacting ligands have predominantly focused on extended planar heteroaromatic scaffolds with the ability to π–π stack onto guanine quartets [19,20,21,22], often equipped with cationic functionalities to enhance affinity and aqueous solubility [23,24]. In contrast, aryl–aryl-connected molecular scaffolds with high rotational flexibility are rather underrepresented as potential G-quadruplex ligands. However, the realization that oligoaryl systems with linear connectivity of their constituent aryl components might prove beneficial in rendering G-quadruplex binders more selective and allowing them to access the diversity regions of G-quadruplexes—previously inaccessible by rigid all-planar binders (i.e., grooves, loops)–has led to recent investigations of such motifs (Figure 1). Among them, pyridyl-oxazole TOxaPy [25], amine-modified pyridyl-oxadiazoles (BOxAzaPy’s) [26] and a phenanthroline-bis-oxazole (Phen2) [27] have emerged as ‘privileged’ recognition motifs. Their nonrigid nature, hydrogen bonding capacity in addition to hydrophobic character, and rotational adaptability towards alternative G-quadruplex surfaces, render them an important addition to the existing toolbox of G-quadruplex ligands. Notably, the molecules previously reported in this class are symmetric to facilitate their synthesis, while their asymmetric counterparts have not been investigated to date, to the best of our knowledge.

The design of some of these compounds follows the ‘head-to-tail’ oxazole connectivity principle, reminiscent of the natural product telomestatin, an oxazole-thiazoline macrocycle with proven success (albeit via a π–π stacking binding mode) in the specific targeting of the telomeric G-quadruplex [28,29]. Given the promising behavior of symmetric pyridine- and oxazole-derived functionalities for G-quadruplex recognition, in the form of either non-macrocyclic synthetic ligands [25,26,27], as well as macrocyclic ones [30], we were inspired to carry out a convergent synthesis and systematic spectroscopic investigation of a new series of relative compounds, putting emphasis on non-symmetric pyridyl–oxazole connectivity. The current effort aimed at expanding our knowledge of how such motifs may contribute to G-quadruplex recognition and selectivity, especially in relation to the size of the oligoaryl compounds and the directionality of their constituent units. The asymmetric nature of these novel *N*,*O*-ligands was anticipated to bode in with the asymmetric environments presented by the DNA targets at hand. The human telomeric sequence and the sequence from the c-Myc oncogene promoter, two G-quadruplex-forming sequences, were selected to test the compounds. Both represent major targets of interest in modern anticancer research and have been studied with the symmetric counterparts previously, which would enable us to draw direct comparisons.

## 2. Results

### 2.1. Synthesis and Purification of a Library of Variable-Length Asymmetric Pyridyl-Oxazole Ligands

**Figure 2:**
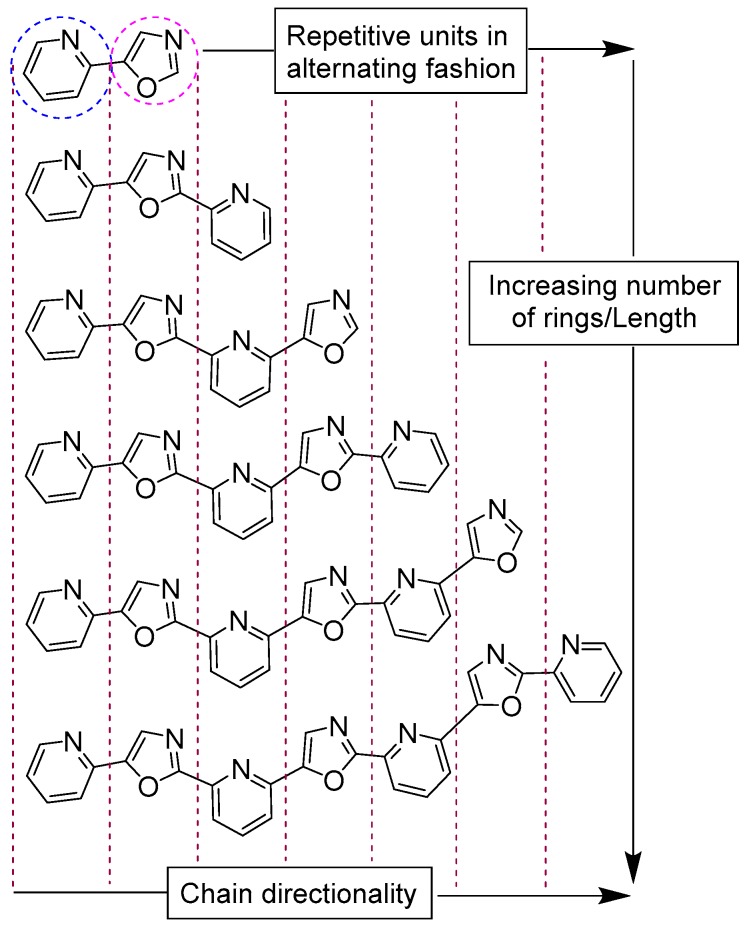
The library of ‘head-to-tail’-connected asymmetric pyridyl-oxazoles discussed in this study. The longer oligomers with 5–7 constituent rings were investigated for G-quadruplex interaction.

Recently developed synthetic methodologies, involving sp^2^ C–H bond activation, combined with cross-coupling reactions [31,32], have potential to revolutionize the production of new and complex oligoaryl *N*,*O*-ligands in a limited number of steps. By employing variations of such methodologies previously described by us [33] and others [25] and compatible with the presence of *N* and *O*-heteroatoms, we have synthesized a small library of pyridyl-oxazole oligomers (with oligomer length varying from 2 to 7 rings) exhibiting a one-directional, ‘head-to-tail’ connectivity of alternating pyridine and oxazole units (Figure 2) [34]. Oligomer directionality is a direct consequence of oxazole orientation within the structures. In this library, we opted for pyridines connected via the 2- or 2,6-positions and oxazoles connected via the 5- or 2,5-positions.

**Scheme 1:**
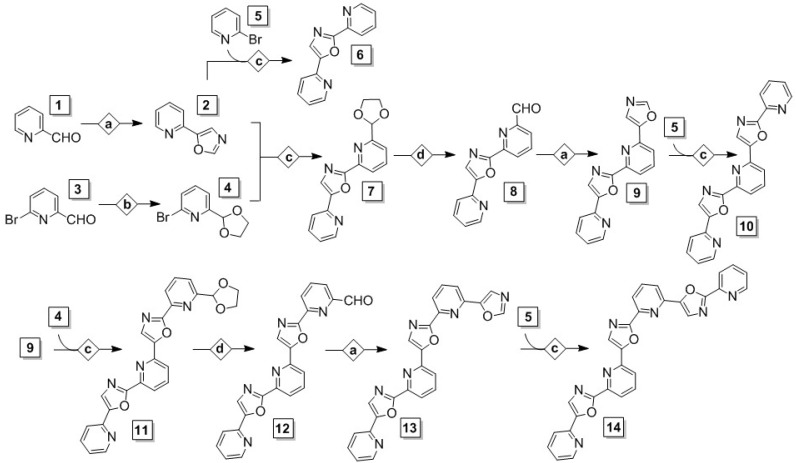
Synthesis of all ‘head-to-tail’-connected pyridyl-oxazole structures discussed in this study, from three initial pyridine-based building blocks and four reaction types. *Conditions*: (**a**) MeOH, TOSMIC, K_2_CO_3_, 65 °C, 4 h; (**b**) Benzene, ethylene glycol, *p*-TsOH (cat.), 100 °C, 24 h; (**c**) 1,4-Dioxane, Cs_2_CO_3_, Pd(OAc)_2_, CuI, Cy_3_P·HBF_4_, 130 °C, 24 h; (**d**) Dimethylsulfoxide/H_2_O (1:1), LiCl, 150 °C, 48 h.

All compounds in this series were derived from three initial substituted pyridine building blocks, as shown in Scheme 1: 2-pyridinecarboxaldehyde (**1**), 6-bromo-2-pyridinecarboxaldehyde (**3**) and 2-bromopyridine (**5**). These are equipped with either aldehyde or bromide functionalities or both, which are necessary for carrying out the desired reaction scheme. Our synthesis relied on two key chemical transformations to construct the oligomers’ skeleton: (I) The conversion of aldehyde moieties to oxazoles in MeOH under Van Leusen conditions (*p*-toluenesulfonyl isocyanate or TOSMIC reagent, in the presence of base) [35] (condition set a in Scheme 1), and (II) A direct C–C cross-coupling to pair pyridine and oxazole, which proceeds via activation of the C–H bond of the 2-position of the oxazole and subsequent coupling to a bromo-substituted position of the pyridine, under Pd(OAc)_2_/CuI cocatalysis [25,33,34] (condition set c). To avoid side reactions during C–C cross-coupling steps, free aldehydes were protected with ethylene glycol as 1,3-dioxolanes [36] (condition set b) and deprotected later with LiCl [37] (condition set d), immediately prior to an oxazole formation step. By appropriate placement of the aldehyde and bromide substituents in the building blocks, our route delivered exclusively the desired ‘head-to-tail’-connected oligomers at the length of 2–7 rings. The process was repetitive, which ensured that longer oligomers could be derived using the shorter ones as precursors.

All intermediates and final products were purified by silica column chromatography before characterization and the three longer oligomers (**10**, **13** and **14**) were further purified by preparative high-performance liquid chromatography (HPLC) on C18-reverse phase silica, prior to any DNA binding studies. The intermediates and final products were characterized by ^1^H and ^13^C nuclear magnetic resonance spectroscopy (^1^H-NMR, ^13^C-NMR) and by matrix-assisted laser desorption/ionization time-of-flight mass spectrometry (MALDI-TOF MS), while UV-Vis spectra were recorded for the longer final oligomers (**10**, **13** and **14**). The purified compounds exhibit satisfactory solubility in several organic solvents, including halogenated, ethyl acetate and dimethylsulfoxide (DMSO). For all DNA studies, they were dissolved in DMSO and diluted with buffer to final solutions of less than 1% DMSO, without indicating any tendency for precipitation.

### 2.2. Determination of Dissociation Constants (K_D_) and Number of Binding Sites (n) for Ligand Interaction with G-Quadruplexes in Fluorescence Titrations

The human telomeric sequence (22AG) and the G-rich sequence from the c-Myc oncogene promoter (Myc2345-Pu22) were chosen to study their interaction with members of this new family of oligoaryl ligands. Guided by docking studies previously described by others for comparable size and type compounds [25,38], the three longer oligomers in this library (**10**, **13** and **14**) were deemed to be of appropriate size for targeting these two G-quadruplex-forming sequences and were, therefore, submitted to fluorescence spectroscopy studies.

Each DNA was prefolded in 10 mM lithium cacodylate buffer (pH 7.2) in the presence of 100 mM monovalent cation (with cloride counterion). It was decided that 22AG be studied in both K^+^- and Na^+^-containing buffer, since it is well established that different morphologies can be obtained under these conditions (hybrid or mixtures vs. a clear antiparallel, respectively) due to the highly dynamic nature of the 22AG sequence [39,40]. In contrast, Myc2345-Pu22 favors a rigid parallel morphology under both conditions [41,42], therefore it was studied in a single (K^+^-rich) buffer.

**Figure 3:**
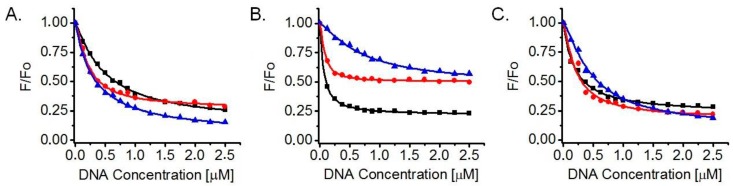
Normalized fluorescence maxima vs. G-quadruplex DNA concentration during fluorescence titrations of ligands **10** (blue triangles), **13** (red circles) and **14** (black squares) with 22AG/K^+^ (**A**); 22AG/Na^+^ (**B**); and Myc2345-Pu22/K^+^ (**C**). Ligand and DNA solutions were prepared in 10 mM lithium cacodylate buffer (pH 7.2), with the monovalent cation at 100 mM concentration.

The synthesized pyridyl-oxazole ligands are intensely fluorescent in aqueous medium in the absence of DNA. Fluorescence titrations were carried out by adding increasing amounts (0–5 equivalents) of prefolded DNA G-quadruplex to a fixed quantity (final concentration 0.5 µM) of each of the studied ligands (**10**, **13** or **14**). Excitation took place at 340 nm, close to the absorption maximum of the ligands, and fluorescence was recorded in the 350–650 nm range. The three oligomers exhibited significant fluorescence reduction upon addition of DNA in all cases (22AG/K^+^, 22AG/Na^+^ and Myc2345-Pu22/K^+^), indicating the onset of interaction (see Supplementary Materials). Normalized fluorescence maxima at different DNA-to-ligand ratios vs. DNA concentration were plotted (Figure 3) and a nonlinear model fitted to the data (growth-sigmoidal/Hill 1, no weights, fixed starting point) led to determination of dissociation constants K_D_ in each case and indicated a 1:1 ligand-to-DNA stoichiometry of binding (number of binding sites *n* = 1) for all ligands, against all tested G-quadruplexes. These results are summarized in Table 1. A statistically insignificant fluorescence reduction (which rendered data-fitting unsuccessful) was observed with the three ligands on a control double-stranded sequence (ds26) in K^+^ buffer (not shown), implying a negligible interaction with this duplex DNA.

### 2.3. Determination of ΔT_m_ Values of G-Quadruplexes Upon Ligand Binding, Based on FRET Melting Assays

To evaluate the effect of ligand binding on the thermodynamic stability of pre-folded G-quadruplexes, a series of Förster resonance energy transfer (FRET) melting assays were carried out by using double-labelled (with FAM and TAMRA dyes, where FAM = 6-carboxyfluorescein and TAMRA = tetramethylrhodamine) single-stranded oligonucleotides F21T (telomeric, in K^+^ and Na^+^) and FmycT (in K^+^) [43]. The measurements were conducted in the absence or presence of a competitive double-stranded DNA sequence, ds26.

**Figure 4:**
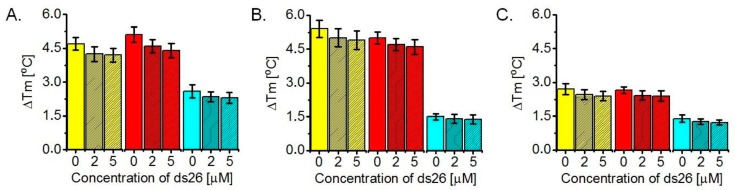
Values of Δ*T*_m_ for ligand interaction with F21T/K^+^ (**A**), F21T/Na^+^ (**B**) and FmycT/K^+^ (**C**), determined via FRET melting assays. Ligands **14** (yellow), **13** (red) and **10** (cyan) were tested at 1 µM final concentration, with the G4-DNA fixed at 0.2 µM and the competing ds26 duplex DNA at 0, 2 or 5 µM. Buffer used was 10 mM lithium cacodylate (pH 7.2), supplemented with: 90 mM LiCl/10 mM KCl in (**A**); 90 mM LiCl/10 mM NaCl in (**B**); and 99 mM LiCl/1 mM KCl in (**C**).

A 10 mM lithium cacodylate buffer (pH 7.2) containing 90 mM LiCl and 10 mM KCl or NaCl was used with the F21T sequence, while a 10 mM lithium cacodylate buffer (pH 7.2) containing 99 mM LiCl and 1 mM KCl was used in the case of FmycT. Ligands **10**, **13** and **14** were tested at 1 µM concentration, with the G-quadruplex-forming DNA at 0.2 µM and competing ds26 DNA at three different concentrations (0, 2 and 5 µM). Excitation took place at 450–495 nm and detection at 515–545 nm. Normalized fluorescence of FAM vs. temperature was plotted and nonlinear fitting to the data (growth-sigmoidal/BiDoseResponse, no weights) allowed determination of DNA melting temperatures in the presence of each ligand, as well as Δ*T*_m_ values (from comparison with the *T*_m_ of the G-quadruplex alone). The determined Δ*T*_m_ values are plotted vs. ds26 concentration in Figure 4. As a control, the double-labelled version of ds26, Fds26T (in K^+^) was also investigated against the ligands by FRET (see Supplementary Data).

### 2.4. Detection of G-Quadruplex Conformational Changes upon Ligand Interaction, via Circular Dichroism (CD) Titrations

We anticipated that rotationally flexible ligands of this type would readily adapt to the unique stereo-environment displayed by each target DNA and promote morphological changes to certain preformed G-quadruplexes. Conformational changes of these chiral entities can be detected by circular dichroism (CD) spectroscopy [44,45,46]. In a preliminary study, we have previously observed that the Myc2345-Pu22 parallel G-quadruplex does not undergo any morphology transition during interaction with ligands of this type, but its pre-existing parallel helical conformation is enhanced [34]. Hence, the current study focuses on the 22AG telomeric sequence, which is considered more labile and polymorphic.

Prior to investigating ligand-DNA mixtures, control ligand-only CD measurements were conducted under K^+^-rich and Na^+^-rich conditions. These revealed that the free ligands are ‘CD-silent’ in their absorbance range, indicating the ligands’ inability to assume helical conformations under the influence of each cation.

**Figure 5:**
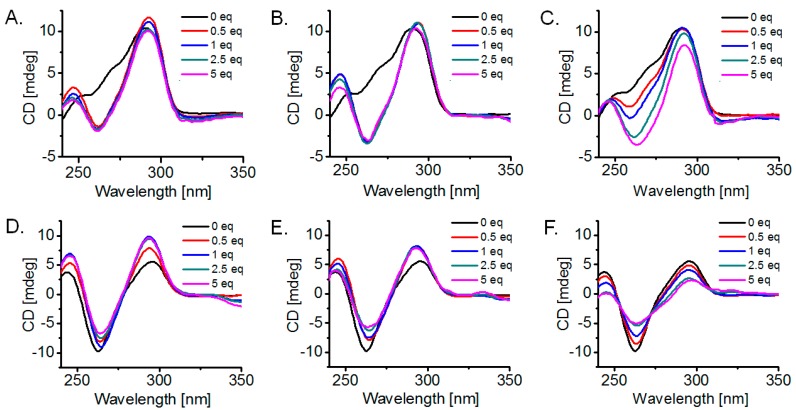
Circular dichroism (CD) titrations of 3 µM prefolded 22AG G-quadruplexes with increasing amounts (0–5 equivalents) of tested ligand: (**A**–**C**). DNA in 10 mM lithium cacodylate buffer (pH = 7.2) with 100 mM KCl, compounds **14**, **13** and **10**, respectively; (**D**–**F**). DNA in 10 mM lithium cacodylate buffer (pH = 7.2) with 100 mM NaCl, compounds **14**, **13** and **10**, respectively.

In CD titrations, increasing amounts of each ligand (0–5 equivalents) were added to 22AG G-quadruplex (DNA final concentration was 3 µM) and the CD spectrum was compared to that of the G-quadruplex in the absence of ligand. The 22AG sequence was prefolded in 10 mM lithium cacodylate buffer (pH = 7.2) in the presence of 100 mM KCl or in the presence of 100 mM NaCl. CD titration curves are presented in Figure 5.

## 3. Discussion

### 3.1. Synthesis

The chemical synthesis of a focused library of oligo-heteroaryl ligands based on the pyridyl-oxazole motif involved a robust and reproducible methodology that utilizes sp^2^ C–H bond activation combined with C–C cross-coupling as a key transformation to create the desired aryl–aryl connectivity between pyridines and oxazoles [34]. The latter have been obtained via a 1,3-dipolar cycloaddition of the TOSMIC reagent (followed by elimination/aromatization), on readily available aldehydes attached to pyridyl building blocks. The repetitive character of the synthesis has facilitated the derivation of longer oligomers from shorter ones in order to allow evaluation of various lengths for recognition of cancer-relevant G-rich DNA sequences 22AG (telomeric) and Myc2345-Pu22 (c-Myc oncogene promoter). This series of ligands differ from previous counterparts in that they are asymmetric and directional with regard to the orientation of their constituent components/rings, which aimed to provide alternative recognition patterns and possibilities upon their interaction with the asymmetric G-quadruplex targets. Notably, this library includes even number-of-rings pyridyl-oxazoles (e.g., **13**), whose symmetric counterparts have not been previously investigated for G-quadruplex recognition.

### 3.2. Ligand Affinity for DNA G-Quadruplexes and Binding Stoichiometry, Based on Fluorescence Titrations

A comparison of the determined dissociation constants for the three ligands (Table 1) reveals a superior binding preference towards the antiparallel telomeric (22AG) conformation stabilized in the presence of Na^+^ for the 7-mer (**14**) (K_D_ = 0.6 × 10^−7^ M^−1^) and 6-mer (**13**) (K_D_ = 0.8 × 10^−7^ M^−1^). Their affinities for this G-quadruplex comparatively to that of the 5-mer (**10**) are 12-fold and 9-fold higher, respectively. This length-dependent trend is consistent with a previous report comparing the affinity of neutral 7-meric vs. 5-meric symmetric pyridyl-oxazoles towards the Na^+^-induced 22AG G-quadruplex [25]. Like the case of symmetric 7-mer TOxaPy, the three asymmetric pyridyl-oxazole ligands studied herein also bind with a 1:1 ligand-to-DNA stoichoimetry. Remarkably, asymmetric ligands **14** and **13** exhibit 3.3 and 2.5 times higher affinity than TOxaPy, which demonstrates a prevalence of the asymmetric over the symmetric pyridyl-oxazole motif in the recognition of this particular antiparallel G-quadruplex. The asymmetric ligands are differentiated from their symmetric counterparts in the following aspects: (i) the actual sequence of pyridine and oxazole units; (ii) the exact placement of *N* and *O* heteroatoms within the structures; and (iii) potentially subtle differences in their rotational conformations. The affinities of **14** and **13** for 22AG/Na^+^ are also comparable to that of related neutral phenanthroline/pyridyl-oxazole-based ligand Phen2 [27].

The K_D_ values for asymmetric ligand interaction with the G-quadruplex conformation of 22AG present under K^+^ conditions reveal that these ligands are not as strongly discriminatory against a K^+^ G-quadruplex as TOxaPy. This different behavior could be attributed to a rearrangement of the initial K^+^ G-quadruplex conformation to one that interacts more favorably with the asymmetric ligands, as indicated by the CD results (Figure 5A–C). It is uncertain whether TOxaPy can induce a similar G-quadruplex rearrangement, since it was not studied by CD. This is unlikely, given its negligible affinity for 22AG in K^+^ [25]. Therefore, we suggest that K_D_ in the asymmetric ligands case refers to a different post-equilibration G-quadruplex morphology than the one encountered by TOxaPy. The interaction with this K^+^ G-quadruplex is weaker than the Na^+^ one for ligands **14** (8-fold) and **13** (3-fold) and stronger for **10** (2-fold). Ligand **13** appears to be the strongest binder for 22AG G-quadruplex in K^+^, with **14** being the weakest of the three ligands against this particular morphology. This ranking is suggestive of dimensional restrictions experienced only by the longest ligand (**14**) upon interaction with the available binding site. Under K^+^ conditions, a 1:1 binding stoichiometry is maintained.

Ligands **13** and **14** have comparable affinities for the Myc2345-Pu22 parallel G-quadruplex in K^+^ buffer (with K_D_ values at 1.7 × 10^−7^ M^−1^ and 1.9 × 10^−7^ M^−1^, respectively, lower than their affinities for the antiparallel 22AG/Na^+^ G-quadruplex), with ligand **10** being a considerably less potent binder for Myc2345-Pu22. A 1:1 stoichiometry was observed in this case as well.

### 3.3. Ligand-Induced Thermodynamic Stabilization of Prefolded G-Quadruplexes, Based on FRET Melting Assays

The Δ*T*_m_ values of the studied G-quadruplexes under the effect of the three oligomeric ligands (Figure 4) are length-dependent. They suggest a comparable and modest thermodynamic stabilization of the K^+^ and Na^+^ telomeric G-quadruplexes (4–6 °C) by ligands **14** and **13**, while in all other cases Δ*T*_m_ values are lower than 3 °C. Given that some of the oligomers exhibit high binding affinities towards both 22AG and Myc2345-Pu22, this weak stabilization would be consistent with a groove or loop binding mode of interaction that does not significantly interfere with the π–π stacking contacts in the core of the G-quadruplexes, which in turn control their overall integrity. For 22AG/Na^+^ (Figure 4B) and Myc2345-Pu22/K^+^ (Figure 4C), the trend correlates well with the observed binding affinities and indicates a dependence of the DNA thermodynamic stabilization on the ligand’s surface area. Anomalous behavior is exhibited by ligand **14** in the 22AG/K^+^ case (Figure 4A), which despite having the lowest binding affinity, induces almost the same stabilization to the DNA as ligand **13** and higher than ligand **10**. This finding suggests that the ability of the ligands to drive DNA conformational transition (in order to reach an energy minimum) rather than their overall affinity for the target is the determining factor under the K^+^ conditions, and in this aspect **14** has an advantage over **10**, as can be seen by CD (Figure 5A,C). The stabilization of 22AG/Na^+^ by **14** and **13** is lower than the one observed for TOxaPy, while the stabilization of 22AG/K^+^ is higher [25].

Critically, none of the three ligands leads to any stabilization of the double-labelled duplex DNA, Fds26T (Δ*T*_m_ ≈ 0 °C at 1 μM ligand concentration, see Supplementary Materials) which, taken together with the minor decrease of Δ*T*_m_ (<15%) in competitive experiments with 10-fold or 25-fold excess of ds26 relative to the G_4_-forming DNA, indicates a significant ligand selectivity in favor of G-quadruplexes and against duplexes that can be further exploited.

### 3.4. G-Quadruplex Conformational Changes Promoted by the Ligands, Based on Circular Dichroism (CD) Study

Significant conformational changes are observed during the CD titration in the K^+^-rich buffer (Figure 5A–C). Under these conditions, the CD signal for the 22AG solution before ligand addition indicates presence of elements corresponding to more than one G-quadruplex morphology, with positive peaks at 250 and 290 nm and a shoulder at 265 nm. It has been previously suggested that in KCl solution, the 22AG sequence may exist either as hybrid [3 + 1]-type form(s) [47] or as a mixture of parallel/hybrid and antiparallel morphologies [47,48], and in these studies the CD profile was identical to the one observed in the current study. Notably, ligands **13** and **14** appear prominent in altering this initial conformational preference and converting it into a seemingly antiparallel conformation, as evidenced by a positive peak at ~295 nm and a negative peak at 260 nm. Similar conformational transitions have been reported only for a handful of other compounds, namely a 5-meric cationic pyridyl-oxadiazole (BOxAzaPy) [26] and isoquinoline alkaloid sanguinarine [49].

Equilibration is fast and is established within 5 min of mixing. Compound **13** induces a more helical conformation compared to **14**, in alignment with its stronger binding. The conformational switch occurs even with 0.5 equivalents of ligand, which suggests the coexistence of two G-quadruplex populations rather than one in the solution prior to ligand addition. Compound **10** requires more ligand equivalents to rearrange the initial structure.

In Na^+^-rich buffer, the inherent preference of the 22AG sequence is clearly for an antiparallel fold. Interaction of compounds **14**, **13** and **10** appears to slightly reduce the helical character of the pre-equilibrium G-quadruplex, but maintains a similar topology, with the most strongly interacting oligomers (**14** and **13**) causing a more obvious effect. Remarkably, the final DNA conformation resembles the one obtained in K^+^, suggesting a strong predisposition of this compound family to drive DNA folding, a property that could be useful in ‘switch-type’ nanodevices.

Notably, the emergence of an induced CD signal above 300 nm (ligand absorption region) in some of the CD titrations indicates that these rotationally versatile ligands are themselves forced into a chiral conformation due to their interaction with the DNA [50].

## 4. Materials and Methods

### 4.1. General

All reactions were performed under an argon atmosphere and anhydrous solvents were used, unless otherwise stated. In flash liquid chromatography purifications, Merck silica gel 60 (0.06–0.2 mm) was used. Final purification of ligands **10**, **13** and **14** was carried out in XBridge BEH C18 OBD column, on a Waters 1525EF semi-preparative HPLC system (Milford, MA, USA), equipped with autosampler, diode array detector and fraction collector. NMR spectra were obtained on a Bruker Avance III Ultrashield Plus spectrometer (Billerica, MA, USA), at 500 MHz for ^1^H-NMR and 125 MHz for ^13^C-NMR, at 25 °C, chemical shifts relative to tetramethylsilane. MS data were collected on a Bruker Autoflex III Smartbeam MALDI-TOF/TOF instrument (Billerica, MA, USA). UV spectra were recorded on a Jenway 6715 UV-Vis Spectrophotometer (Stone, UK). All DNA sequences used were purchased from Eurogentec (Liège, Belgium) as synthetic oligonucleotides, purified by HPLC and dialysis. Graphs were constructed and data fitting was carried out in OriginPro 8.0 (Northampton, MA, USA).

### 4.2. Synthetic Methods

#### 4.2.1. General Method for Conversion of Aldehyde to Oxazole (Condition Set a)

In a round-bottom flask containing dry MeOH (10 mL/mmol of aldehyde) were added the aldehyde (1 equiv.), TOSMIC reagent (1.1 equiv.) and K_2_CO_3_ (2.2 equiv.), in this order. The flask was fitted with a vertical condenser and the mixture was refluxed at 65 °C for 4 h. The solvent was entirely removed under reduced pressure and the residue was resuspended in EtOAc and stirred for 30 min at room temperature. Filtration led to removal of insoluble materials and the solution was concentrated under reduced pressure and applied to a silica column for flash chromatography. Elution took place by using either a hexane/EtOAc step gradient (from 2:1 to 1:2 to pure EtOAc) or a CH_2_Cl_2_/MeOH step gradient (from 99:1 to 90:10), depending on product polarity. This method was applied to the synthesis of compounds **2**, **9** and **13**.

*5-(Pyridin-2-yl)oxazole (**2**): Reaction scale 20 mmol of aldehyde* **1**; Chromatography with hexane/EtOAc step gradient; Product yellow oil; Yield 98%; ^1^H-NMR (CDCl_3_): *δ* (ppm) = 7.19 (1H, ddd, *J*_1_ = 7.8 Hz, *J*_2_ = 4.7 Hz, *J*_3_ = 1.0 Hz), 7.61 (1H, d, *J* = 7.8 Hz), 7.65 (1H, s), 7.71 (1H, dt, *J*_1_ = 7.8 Hz, *J*_2_ = 1.8 Hz), 7.93 (1H, s), 8.58 (1H, d, *J* = 5.5 Hz); ^13^C-NMR (CDCl_3_): *δ* (ppm) = 119.0, 122.7, 124.5, 136.6, 146.7, 149.5, 150.7, 150.8; MS (MALDI-TOF): *m*/*z* = 147.23 [M + H]^+^ (calcd. for C_8_H_6_N_2_O: 146.05).

*2-[6-(Oxazol-5-yl)pyridin-2-yl]-5-(pyridin-2-yl)oxazole* (**9**): Reaction scale 1 mmol of aldehyde **8**; Chromatography with hexane/EtOAc step gradient; Product white solid; Yield 68%; ^1^H-NMR (CDCl_3_): *δ* (ppm) = 7.28 (1H, ddd, *J*_1_ =7.6 Hz, *J*_2_ = 4.7 Hz, *J*_3_ = 1.0 Hz), 7.76 (1H, d, *J* = 7.8 Hz), 7.83 (1H, dt, *J*_1_ = 7.6 Hz, *J*_2_ = 1.5 Hz), 7.89 (1H, d, *J* = 8.1 Hz), 7.92 (3H, m, signals overlapping), 8.02 (1H, s), 8.15 (1H, d, *J* = 8.1 Hz), 8.67 (1H, d, *J* = 4.7 Hz); ^13^C-NMR (CDCl_3_): *δ* (ppm) = 119.8, 120.3, 121.7, 123.3, 125.9, 127.3, 137.1, 138.0, 146.1, 146.9, 147.6, 149.9, 150.5, 151.4, 152.0, 160.2; MS (MALDI-TOF): *m*/*z* = 291.18 [M + H]^+^ (calcd. for C_16_H_10_N_4_O_2_: 290.08).

*2-[6-(Oxazol-5-yl)pyridin-2-yl]-5-{6-[5-(pyridin-2-yl)oxazol-2-yl]-pyridine-2-yl}oxazole* (**13**): Reaction scale 0.15 mmol of aldehyde **12**; Chromatography with CH_2_Cl_2_/MeOH step gradient; Product white solid; Yield 42%; ^1^H-NMR (CDCl_3_): *δ* (ppm) = 7.32 (1H, dd, *J*_1_ = 5.9 Hz, *J*_2_ = 3.4 Hz), 7.80 (1H, d, *J* = 7.7 Hz), 7.92 (1H, app. t, *J* = 7.7 Hz), 7.96–8.00 (4H, m, signals overlapping), 8.02–8.06 (3H, m, signals overlapping), 8.11 (1H, s), 8.22 (1H, d, *J* = 7.7 Hz), 8.70 (1H, d, *J* = 4.5 Hz); ^13^C-NMR (CDCl_3_): *δ* (ppm) = 120.0, 120.5, 120.8, 121.8, 121.9, 123.4, 125.7, 126.3, 127.5, 128.8, 130.9, 132.4, 137.4, 137.9, 138.0, 146.0, 146.1, 146.8, 146.9, 147.4, 147.6, 149.7, 160.3, 160.8; MS (MALDI-TOF): *m*/*z* = 435.26 [M + H]^+^ (calcd. for C_24_H_14_N_6_O_3_: 434.11); UV (DMSO): *λ*_1_ = 263 nm (ε_1_ = 1.4 × 10^4^ L/(mol.cm)), *λ*_2_ = 303 nm (ε_2_ = 2.5 × 10^4^ L/(mol.cm)), *λ*_3_ = 333 nm (ε_3_ = 2.3 × 10^4^ L/(mol.cm)).

#### 4.2.2. General Method for Conversion of Aldehyde to 1,3-Dioxolane (Condition Set b)

In a round-bottom flask, aldehyde (1 equiv.), ethylene glycol (2 equiv.) and *p*-toluenesulfonic acid monohydrate (0.05 equiv.) were dissolved in dry benzene (5 mL/mmol of aldehyde). The flask was fitted with a Dean–Stark apparatus and reflux took place at 100 °C for 24 h. The mixture was then cooled to room temperature and 1% (*w*/*w*) aqueous Na_2_CO_3_ was added to quench. The mixture was transferred to a separatory funnel and extracted with CH_2_Cl_2_ (3×). The combined organic extract was dried over Na_2_SO_4_, and the solvent was removed under reduced pressure. The crude product was redissolved in CH_2_Cl_2_ and applied to a silica column for flash chromatography. Elution took place by using a CH_2_Cl_2_/MeOH step gradient (from 95:5 to 90–10). This method was applied to the synthesis of compound **4**.

*2-Bromo-6-(1,3-dioxolan-2-yl)pyridine* (**4**): Reaction scale 5 mmol of aldehyde **3**; Chromatography with CH_2_Cl_2_/MeOH step gradient; Product yellow oil; Yield 96%; ^1^H-NMR (CDCl_3_): *δ* (ppm) = 4.07 (2H, m), 4.16 (2H, m), 5.81 (1H, s), 7.47 (1H, d, *J* = 7.8 Hz), 7.50 (1H, d, *J* = 7.8 Hz), 7.59 (1H, app. t, *J* = 7.8 Hz); ^13^C-NMR (CDCl_3_): *δ* (ppm) = 65.6, 102.8, 119.4, 128.5, 139.1, 141.7, 158.5; MS (MALDI-TOF): *m*/*z* = 230.06 [M + H]^+^ (calcd. for C_8_H_8_BrNO_2_: 228.97).

#### 4.2.3. General Method for Oxazole C–H Activation/C–C Cross-Coupling to Bromopyridine (Condition Set c)

A round-bottom flask was charged with oxazole (1 equiv.), (substituted or unsubstituted) bromopyridine (1 equiv.), Cs_2_CO_3_ (2.2 equiv.), Pd(OAc)_2_ (0.2 equiv.), CuI (1.1 equiv.) and Cy_3_P·HBF_4_ (0.1 equiv.). The flask was fitted with a vertical condenser and was set under argon atmosphere. Anhydrous 1,4-dioxane (20 mL/mmol of oxazole) was added by syringe, and the mixture was refluxed at 130 °C for 24 h. The mixture was then cooled to room temperature and filtered through a sintered Buchner funnel to remove insoluble materials. The dioxane solution was then dried under reduced pressure. The crude residue was redissolved in a small amount of dichloromethane and applied to a silica gel column for flash chromatography. Elution took place by using CH_2_Cl_2_/MeOH (95:5) or CH_2_Cl_2_/MeOH step gradient (from 99:1 to 90:10) or EtOAc/MeOH step gradient (from 99:1 to 95:5), depending on product polarity and reaction mixture complexity. This method was applied to the synthesis of compounds **6**, **7**, **10**, **11** and **14**.

*2,5-Di(pyridin-2-yl)oxazole* (**6**): Reaction scale 3.5 mmol of oxazole **2** and 3.5 mmol of bromopyridine **5**; Chromatography with CH_2_Cl_2_/MeOH step gradient; Product pale yellow solid; Yield 46%; ^1^H-NMR (CDCl_3_): *δ* (ppm) = 7.23 (1H, app. t, *J* = 6.2 Hz), 7.37 (1H, app. t, *J* = 6.0 Hz), 7.76 (1H, dt, *J*_1_ = 7.6 Hz, *J*_2_ = 1.7 Hz), 7.79–7.89 (2H, m, signals overlapping), 7.88 (1H, s), 8.20 (1H, d, *J* = 8.0 Hz), 8.63 (1H, d, *J* = 4.5 Hz), 8.75 (1H, d, *J* = 4.5 Hz); ^13^C-NMR (CDCl_3_): *δ* (ppm) = 119.5, 122.2, 123.0, 124.6, 126.9, 136.6, 136.7, 145.7, 146.8, 149.7, 149.8, 151.7, 160.4; MS (MALDI-TOF): *m*/*z* = 224.12 [M + H]^+^ (calcd. for C_13_H_9_N_3_O: 223.07).

*2-[6-(1,3-Dioxolan-2-yl)pyridin-2-yl]-5-(pyridin-2-yl)oxazole* (**7**): Reaction scale 1 mmol of oxazole **2** and 1 mmol of bromopyridine **4**; Chromatography with CH_2_Cl_2_/MeOH (95:5); Product white solid; Yield 53%; ^1^H-NMR (CDCl_3_): *δ* (ppm) = 4.10 (2H, t, *J* = 7.0 Hz), 4.20 (2H, t, *J* = 7.0 Hz), 5.97 (1H, s), 7.22 (1H, app. t, *J* = 6.0 Hz), 7.63 (1H, d, *J* = 7.8 Hz), 7.76 (1H, app. t, *J* = 7.5 Hz), 7.85–7.87 (3H, m, signals overlapping), 8.18 (1H, d, *J* = 8.4 Hz), 8.62 (1H, d, *J* = 4.8 Hz); ^13^C-NMR (CDCl_3_): *δ* (ppm) = 65.6, 103.5, 119.7, 121.7, 122.6, 123.1, 127.1, 136.8, 137.7, 145.4, 147.0, 149.9, 151.9, 157.9, 160.5; MS (MALDI-TOF): *m*/*z* = 296.28 [M + H]^+^ (calcd. for C_16_H_13_N_3_O_3_: 295.10).

2*-(Pyridin-2-yl)-5-{6-[5-(pyridin-2-yl)oxazol-2-yl]pyridin-2-yl}-oxazole* (**10**): Reaction scale 0.25 mmol of oxazole **9** and 0.25 mmol of bromopyridine **5**; Chromatography with CH_2_Cl_2_/MeOH step gradient; Product pale yellow solid; Yield 46%; ^1^H-NMR (CDCl_3_): *δ* (ppm) = 7.29 (1H, ddd, *J*_1_ = 7.9 Hz, *J*_2_ = 4.7 Hz, *J*_3_ = 1.0 Hz), 7.42 (1H, app. t, *J* = 6.0 Hz), 7.84 (1H, dt, *J*_1_ = 7.8 Hz, *J*_2_ = 1.6 Hz), 7.87 (1H, app. t, *J* = 7.7 Hz), 7.92 (1H, s), 7.93 (1H, d, *J* = 7.0 Hz), 7.95 (1H, app. t, *J* = 7.7 Hz), 7.99 (1H, d, *J* = 7.7 Hz), 8.11 (1H, s), 8.17 (1H, dd, *J*_1_ = 7.7 Hz, *J*_2_ = 0.9 Hz), 8.26 (1H, d, *J* = 7.7 Hz), 8.68 (1H, d, *J* = 4.7 Hz), 8.80 (1H, d, *J* = 3.4 Hz); ^13^C-NMR (CDCl_3_): *δ* (ppm) = 119.8, 120.6, 121.7, 122.6, 123.3, 125.0, 127.2, 128.3, 136.9, 137.0, 137.9, 145.8, 146.1, 147.0, 147.5, 150.0, 150.1, 151.4, 152.1, 160.2, 160.8; MS (MALDI-TOF): *m*/*z* = 368.19 [M + H]^+^ (calcd. for C_21_H_13_N_5_O_2_: 367.11); UV (DMSO): *λ*_1_ = 265 nm (ε_1_ = 1.5 × 10^4^ L/(mol.cm)), *λ*_2_ = 305 nm (ε_2_ = 2.4 × 10^4^ L/(mol.cm)), *λ*_3_ = 335 nm (ε_3_ = 2.2 × 10^4^ L/(mol.cm)).

*2-[6-(1,3-Dioxolan-2-yl)pyridin-2-yl]-5-{6-[5-(pyridin-2-yl)oxazol-2-yl]pyridin-2-yl}oxazole* (**11**): Reaction scale 0.2 mmol of oxazole **9** and 0.2 mmol of bromopyridine **4**; Chromatography with EtOAc/MeOH step gradient; Product white solid; Yield 43%; ^1^H-NMR (CDCl_3_): *δ* (ppm) = 4.14 (2H, t, *J* = 6.2 Hz), 4.24 (2H, t, *J* = 6.2 Hz), 6.02 (1H, s), 7.29 (1H, app. t, *J* = 6.0 Hz), 7.69 (1H, d, *J* = 7.8 Hz), 7.85 (1H, app. t, *J* = 7.8 Hz), 7.91–7.99 (5H, m, signals overlapping), 8.11 (1H, s), 8.18 (1H, d, *J* = 7.5 Hz), 8.25 (1H, d, *J* = 8.0 Hz), 8.68 (1H, d, *J* = 3.7 Hz); ^13^C-NMR (CDCl_3_): *δ* (ppm) = 65.8, 103.5, 119.8, 120.7, 121.7, 121.9, 122.9, 123.3, 127.2, 128.3, 137.0, 137.8, 137.9, 145.4, 146.1, 147.1, 147.5, 150.0, 151.4, 152.1, 157.9, 160.2, 160.6 ppm; MS (MALDI-TOF): *m*/*z* = 440.20 [M + H]^+^ (calcd. for C_24_H_17_N_5_O_4_: 439.13).

*2-(Pyridin-2-yl)-5-[6-(5-{6-[5-(pyridin-2-yl)oxazol-2-yl]pyridin-2-yl}oxazol-2-yl)pyridin-2-yl]oxazole* (**14**): Reaction scale 0.1 mmol of oxazole **13** and 0.1 mmol of bromopyridine **5**; Chromatography with CH_2_Cl_2_/MeOH step gradient; Product white solid; Yield 44%; ^1^H-NMR (CDCl_3_): *δ* (ppm) = 7.30 (1H, dd, *J*_1_ = 7.8 Hz, *J*_2_ = 5.4 Hz), 7.43 (1H, bs), 7.89 (2H, app. t, *J* = 7.6 Hz), 7.94 (1H, s), 7.97–8.03 (5H, m, signals overlapping), 8.13 (1H, s), 8.17 (1H, bs), 8.21 (2H, m, signals overlapping), 8.28 (1H, d, *J* = 5.4 Hz), 8.69 (1H, d, *J* = 4.6 Hz), 8.81 (1H, bs); ^13^C-NMR (CDCl_3_): *δ* (ppm) = 119.6, 119.7, 120.2, 121.5, 122.3, 123.1, 123.2, 124.7, 125.9, 127.1, 127.2, 136.8, 136.9, 137.9, 145.9, 146.0, 146.9, 147.0, 147.4, 149.7, 149.8, 149.9, 150.0, 150.4, 151.3, 151.8, 152.0, 160.0, 160.5; MS (MALDI-TOF): *m*/*z* = 512.17 [M + H]^+^ (calcd. for C_29_H_17_N_7_O_3_: 511.14); UV (DMSO): *λ*_1_ = 262 nm (ε_1_ = 1.6 × 10^4^ L/(mol.cm)), *λ*_2_ = 302 nm (ε_2_ = 2.6 × 10^4^ L/(mol.cm)), *λ*_3_ = 333 nm (ε_3_ = 2.4 × 10^4^ L/(mol.cm)).

#### 4.2.4. General Method for Conversion of 1,3-Dioxolane to Aldehyde (Condition Set d)

A round-bottom flask was charged with 1,3-dioxolane (1 equiv.) and DMSO (6 mL/mmol of dioxolane). A solution of LiCl (10 equiv.) in water (6 mL/mmol of dioxolane) was added, and a vertical condenser was fitted to the flask. The mixture was refluxed at 150 °C for 48 h and then cooled to room temperature, diluted with water, and extracted with CH_2_Cl_2_ (3×). The organic extracts were combined and dried over Na_2_SO_4_, and the solvent was removed under reduced pressure. The crude product was redissolved in CH_2_Cl_2_ and applied to a silica gel column for flash chromatography. It was eluted by using either a hexane/EtOAc step gradient (from 1:1 to 1:3) or a EtOAc/MeOH step gradient (from 99:1 to 95:5), depending on product polarity. This method was applied to the synthesis of compounds **8** and **12**.

*6-[5-(Pyridin-2-yl)oxazol-2-yl]picolinaldehyde* (**8**): Reaction scale 1.5 mmol of dioxolane **7**; Chromatography with hexane/EtOAc step gradient; Product white solid; Yield 67%; ^1^H-NMR (CDCl_3_): *δ* (ppm) = 7.28 (1H, ddd, *J*_1_ = 7.6 Hz, *J*_2_ = 4.8 Hz, *J*_3_ = 1.2 Hz), 7.81 (1H, dt, *J*_1_ = 7.6 Hz, *J*_2_ = 1.7 Hz), 7.87 (1H, td, *J*_1_ = 7.8 Hz, *J*_2_ = 1.0 Hz), 7.90 (1H, s), 8.03 (2H, m, signals overlapping), 8.40 (1H, dd, *J*_1_ = 5.7 Hz, *J*_2_ = 3.2 Hz), 8.66 (1H, dt, *J*_1_ = 4.9 Hz, *J*_2_ = 1.0 Hz), 10.23 (1H, s); ^13^C-NMR (CDCl_3_): *δ* (ppm) = 119.9, 122.2, 123.5, 126.2, 127.4, 137.1, 138.1, 146.4, 146.8, 150.0, 152.3, 153.0, 159.7, 193.0; MS (MALDI-TOF): *m*/*z* = 252.18 [M + H]^+^ (calcd. for C_14_H_9_N_3_O_2_: 251.07).

*6-(5-{6-[5-(Pyridin-2-yl)oxazol-2-yl]pyridin-2-yl}oxazol-2-yl)-picolinaldehyde* (**12**): Reaction scale 0.2 mmol of dioxolane **11**; Chromatography with EtOAc/MeOH step gradient; Product pale yellow solid; Yield 80%; ^1^H-NMR (CDCl_3_): *δ* (ppm) = 7.35 (1H, app. t, *J* = 6.3 Hz), 7.91 (1H, app. t, *J* = 7.3 Hz), 7.96–8.02 (3H, m, signals overlapping), 8.03–8.09 (3H, m, signals overlapping), 8.13 (1H, s), 8.22 (1H, dd, *J*_1_ = 5.5 Hz, *J*_2_ = 3.5 Hz), 8.46 (1H, app. t, *J* = 4.4 Hz), 8.70 (1H, d, *J* = 4.4 Hz), 10.25 (1H, s); ^13^C-NMR (CDCl_3_): *δ* (ppm) = 120.1, 120.8, 122.0, 122.1, 122.4, 123.5, 125.3, 126.3, 128.4, 138.1, 138.2, 138.3, 146.0, 146.3, 147.2, 149.1, 151.7, 153.0, 155.5, 159.9, 160.4, 192.9; MS (MALDI-TOF): *m*/*z* = 396.15 [M + H]^+^ (calcd. for C_22_H_13_N_5_O_3_: 395.10).

### 4.3. Fluorescence Titrations

A 10 mM lithium cacodylate buffer (pH 7.2) containing either KCl or NaCl at 100 mM concentration was used for preparing all work solutions. Sequences 22AG (telomeric, 5′-AGGGTTAGGGTTAGGGTTAGGG-3′) and Myc2345-Pu22 (c-Myc, 5′-TGAGGGTGGGTAGGGT GGGTAA-3′) were employed. DNA stock solution of 100-µM concentration in MilliQ water was diluted with buffer to produce a 5-µM DNA work solution. This buffered DNA solution was annealed at 95 °C for 5 min, then allowed to cool slowly to room temperature overnight. Starting from 4 mM ligand stock solutions in DMSO, 20 μM ligand work solutions in buffer were prepared. Fifteen mixtures representing various DNA-to-ligand molar ratios in the titration (0, 0.25, 0.5, 0.75, 1, 1.25, 1.5, 1.75, 2, 2.5, 3, 3.5, 4, 4.5, 5) were prepared, by combining appropriate volumes of the above-mentioned work solution of ligand, work solution of DNA and additional buffer, all to the same final volume and to a fixed final concentration of tested ligand (0.5 µM), but variable final concentration of DNA. The emission spectrum of each mixture was recorded on a Jasco FP-6300 spectrofluorometer (Easton, MD, USA.). All spectra were obtained at r.t. by using a high-precision quartz suprasil cuvette (light path 10 mm × 0.5 mm). Excitation took place at 340 nm and emission spectra were recorded in the 350–650 nm range, with the following parameters: 5 nm or 10 nm bandwidth (exc.), 5 nm or 10 nm bandwidth (em.), medium response, high sensitivity, 0.5 nm data pitch, 1000 nm/min^−1^ scanning speed, and three accumulations. Correction was made to all spectra by subtracting the spectrum of the buffer, containing the same percentage of DMSO as the mixtures. Emission maxima of the mixtures (F) were normalized by dividing the emission maximum of the ligand in the absence of DNA (F_o_). Normalized emission maxima (F/F_o_) were plotted vs. DNA concentration and the data was fitted with a nonlinear model (growth-sigmoidal/Hill1, no weights, fixed starting point), which allowed determination of dissociation constant (K_D_) and number of binding sites (*n*) in each titration.

### 4.4. FRET Melting Assays

Sequences F21T (telomeric, 5′-FAM-GGGTTAGGGTTAGGGTTAGGG-TAMRA-3′) and FmycT (Myc promoter, 5′-FAM-TTGAGGGTGGGTAGGGTGGGTAA-TAMRA-3′) were employed, as well as dye-free ds26 (5′-CAATCGGATCGAATTCGATCCGATTG-3′), a self-hybridization duplex for inclusion in competitive experiments. A 10 mM lithium cacodylate buffer (pH 7.2) that contained LiCl at 90 mM and either KCl or NaCl at 10 mM concentration was used for preparing work solutions of F21T. A 10 mM lithium cacodylate buffer (pH 7.2) containing LiCl at 99 mM and KCl at 1 mM concentration was used for preparing work solutions of FmycT. Double-labelled DNA with FAM and TAMRA dyes was initially prepared as a 20 µM stock solution in MilliQ water, then diluted with appropriate buffer to a 0.6 µM work solution. This solution was annealed at 95 °C for 5 min, then allowed to cool slowly to room temperature overnight. Each ligand was used from a stock solution of 4 mM concentration in DMSO and diluted to obtain a work solution of 3 µM concentration, using the same buffer as for the DNA under study. From a 1-mM stock solution of ds26 in MilliQ water, two work solutions of 6 µM and 15 µM in buffer were prepared (concentrations based on mol of double helix). Ligand-G_4_ DNA mixtures were prepared in the wells of a 96-well PCR plate (Biorad Laboratories, Hercules, CA, USA) by mixing 13 µL of 0.6 µM DNA solution and 13 µL of 3 µM ligand solution. Finally, 13 µL of buffer or 13 µL of ds26 solution (either 6 µM or 15 µM) was added in each well to reach a final volume of 39 µL. This afforded a fixed final G_4_ DNA concentration (0.2 µM) in each well, fixed ligand concentration (1 µM) and a variable ds26 duplex concentration (0, 2 or 5 µM). Controls that contained only G_4_ DNA at the same concentration as the mixtures were included in the plate. Measurements were performed on a PCR Stratagene Mx3005P (Agilent Technologies, Santa Clara, CA, USA) with excitation at 450–495 nm and detection at 515–545 nm. Readings were taken from 25 °C to 95 °C at intervals of 0.5 °C, maintaining a constant temperature for 30 s before each reading. Each measurement was done in triplicate. Lowest and highest FAM fluorescence were set at 0 and 1 respectively for normalization, and the normalized fluorescence was plotted vs. temperature. The data was fitted with a nonlinear model (growth-sigmoidal/BiDoseResponse, no weights), which allowed determination of *T*_m_ values of the G_4_ DNA in the presence and absence of ligand, and in presence of various concentrations of competing ds26. The same experiment was modified, to allow determination of Δ*T*_m_ in the case of double-labelled duplex Fds26T under noncompetitive conditions.

### 4.5. Circular Dichroism (CD) Titrations

The telomeric sequence 22AG (5′-AGGGTTAGGGTTAGGGTTAGGG-3′) was used. A work solution of 100 µM DNA in 10-mM lithium cacodylate buffer (pH 7.2) containing either KCl or NaCl at 100 mM concentration was annealed at 95 °C for 5 min and then cooled down to room temperature slowly overnight. Starting from 4 mM ligand stock solutions in DMSO, 40 μM ligand work solutions in buffer were prepared. Five mixtures representing various ligand-to-DNA molar ratios in the titration (0, 0.5, 1, 2.5 and 5) were prepared, by combining appropriate volumes of the above-mentioned work solution of DNA, work solution of ligand, DMSO and additional buffer, all to the same final volume and to a fixed final concentration of DNA (3 μM), but variable final concentration of ligand. The DMSO percentage was the same in all mixtures, including the ones lacking ligand. Mixing took place for 5–10 min prior to CD measurements. The spectra were recorded on a Jasco J-815 spectrometer (Easton, MD, USA). All CD spectra were obtained at 21 °C by using quartz curvette (light path 10 mm × 10 mm). The spectra were recorded in the 240–350 nm range, with the following parameters: standard sensitivity, 1 s D.I.T., 2 nm bandwidth, 1 nm data pitch, 50 nm min^−1^ scanning speed, and three accumulations. The scan of the buffer was subtracted from the scan of each sample. The CD signal was plotted vs. wavelength for each mixture.

## 5. Conclusions

Obtaining ligands with high affinities and intrinsic selectivities for G-quadruplex vs. duplex DNA or discrimination for a specific G-quadruplex morphology over others remain a challenge in the G-quadruplex field, especially in light of the potential utility of these structures for anticancer purposes. As part of an effort to identify such ligands, a series of ‘head-to-tail’-connected asymmetric pyridyl-oxazoles were developed and evaluated against 22AG and Myc2345-Pu22 G-quadruplexes. By design, these molecules represent novel entries into a class of rotationally flexible oligoaryl compounds that, due to their limited rigidity and adaptability to the target surface and multivalent bonding capacity, may be well-suited to target alternative sites of G-quadruplexes from those exploited by the more conventional intercalator-type ligands.

The study examined the effect of asymmetric rather than symmetric connectivity of the ligands’ constituent units, as well as the effect of ligand length, and the consequences of these parameters for target affinity, target stabilization and target conformational modification. It is established herein that the neutral asymmetric pyridyl-oxazole motif, particularly when consisting of a minimum of six alternating pyridine and oxazole units, serves as a potent binding element for recognition of G-quadruplexes, especially the antiparallel 22AG/Na^+^ one, for which it indicates improved binding affinity compared to known symmetric counterparts [25]. The length-dependence of the binding offers a tool for probing the ‘span’ of the binding site. The same motif demonstrates satisfactory (albeit lower) affinity for 22AG/K^+^ and Myc2345-Pu22/K^+^ as well which, appropriately embedded in the context of a second-generation of ligands, could be fine-tuned to afford elevated discriminatory ability between these diverse G-quadruplex morphologies and lead to specialized sensors for DNA secondary structures. While being modest stabilizers of pre-existing 22AG G-quadruplexes, these molecules could be further optimized in this regard by employing the strategy of amino/cationic side-arms appendage. This has been demonstrated before for BOxAzaPy’s [26] in order to render them more competent as G-quadruplex stabilizers. Interestingly, the competition FRET melting results provide evidence that the long-standing problem of G-quadruplex vs. duplex selectivity can be bypassed with this family of ligands. Finally, the longer ligands demonstrate a remarkable ability to induce a sharp conformational transition to the 22AG/K^+^ quadruplex, which could be exploited in bioinspired ‘switch-type’ nanodevices.

## Figures and Tables

**Table 1:** Dissociation constant (K_D_, in M^−1^) values and number of binding sites (*n*), as determined by nonlinear curve fitting (growth-sigmoidal/Hill 1, no weights, fixed starting point) of fluorescence titrations data. In all cases, adjusted R^2^ was ≥ 0.995.

Ligand	22AG/K^+^	22AG/Na^+^	Myc2345-Pu22/K^+^
**10**	K_D_ = (3.2 ± 0.1) × 10^−7^	K_D_ = (7.2 ± 0.8) × 10^−7^	K_D_ = (4.8 ± 0.3) × 10^−7^
	*n* = 1.0 ± 0.0	*n* = 1.3 ± 0.1	*n* = 1.3 ± 0.1
**13**	K_D_ = (2.2 ± 0.1) × 10^−7^	K_D_ = (0.8 ± 0.1) × 10^−7^	K_D_ = (1.7 ± 0.1) × 10^−7^
	*n* = 1.3 ± 0.1	*n* = 1.4 ± 0.2	*n* = 1.2 ± 0.1
**14**	K_D_ = (5.0 ± 0.3) × 10^−7^	K_D_ = (0.6 ± 0.0) × 10^−7^	K_D_ = (1.9 ± 0.1) × 10^−7^
	*n* = 1.1 ± 0.1	*n* = 1.1 ± 0.1	*n* = 1.0 ± 0.1

## Supplementary Materials

The following are available online: ^1^H-NMR and ^13^C-NMR spectra; Fluorescence titration curves; FRET melting temperature curves; Δ*T*_m_ for ligand binding to Fds26T.
