# Supplementary material for: Investigation of ‘Head-to-Tail’-Connected Oligoaryl N,O-Ligands as Recognition Motifs for Cancer-Relevant G-Quadruplexes
Source: Molecules. 2017 Dec 6;22(12):2160. doi: 10.3390/molecules22122160 (PMC6149995; doi:10.3390/molecules22122160)
Supplement: Supplementary file 1 [file molecules-22-02160-s001.pdf]

Investigation of ‘Head-to-Tail’-Connected Oligoaryl *N*,*O*-Ligands as Recognition Motifs for Cancer-Relevant G-Quadruplexes

Natalia Rizeq [1] and Savvas N. Georgiades [1,*]

[1] Department of Chemistry, University of Cyprus, 1 Panepistimiou Avenue, Aglandjia, 2109, Nicosia, Cyprus
***** Correspondence: georgiades.savvas@ucy.ac.cy; Tel.: +357-22-892779

Supplementary Materials

Table of Contents

- ^{1}H and ^{13}C NMR spectra S-2
- Fluorescence titration curves S-13
- FRET melting temperature curves S-16
- ΔT_m for Fds26T S-22

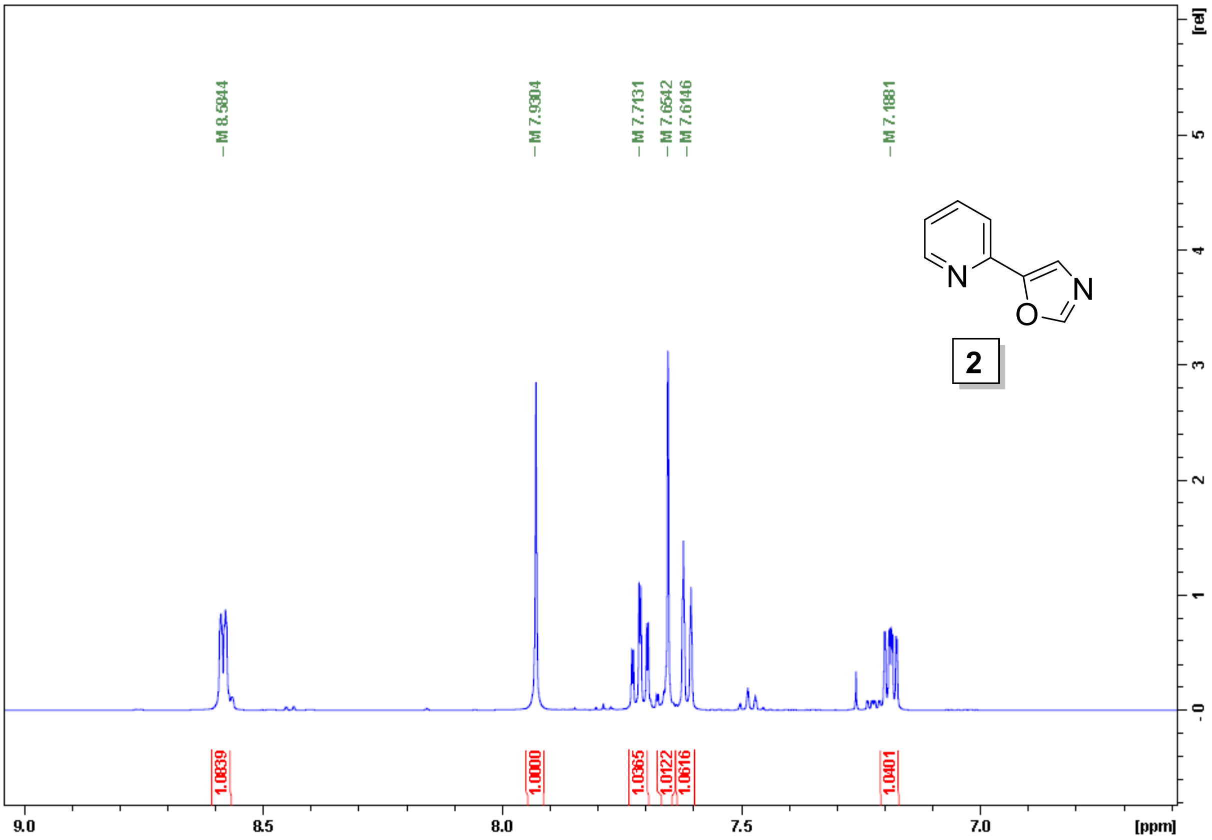
M 8.5844
M 7.9304
M 7.7131
M 7.6542
M 7.6146
M 7.1881
1.0839
1.0000
1.0365
1.0122
1.0616
1.0401
2
N
O
N
[rel]
[ppm]

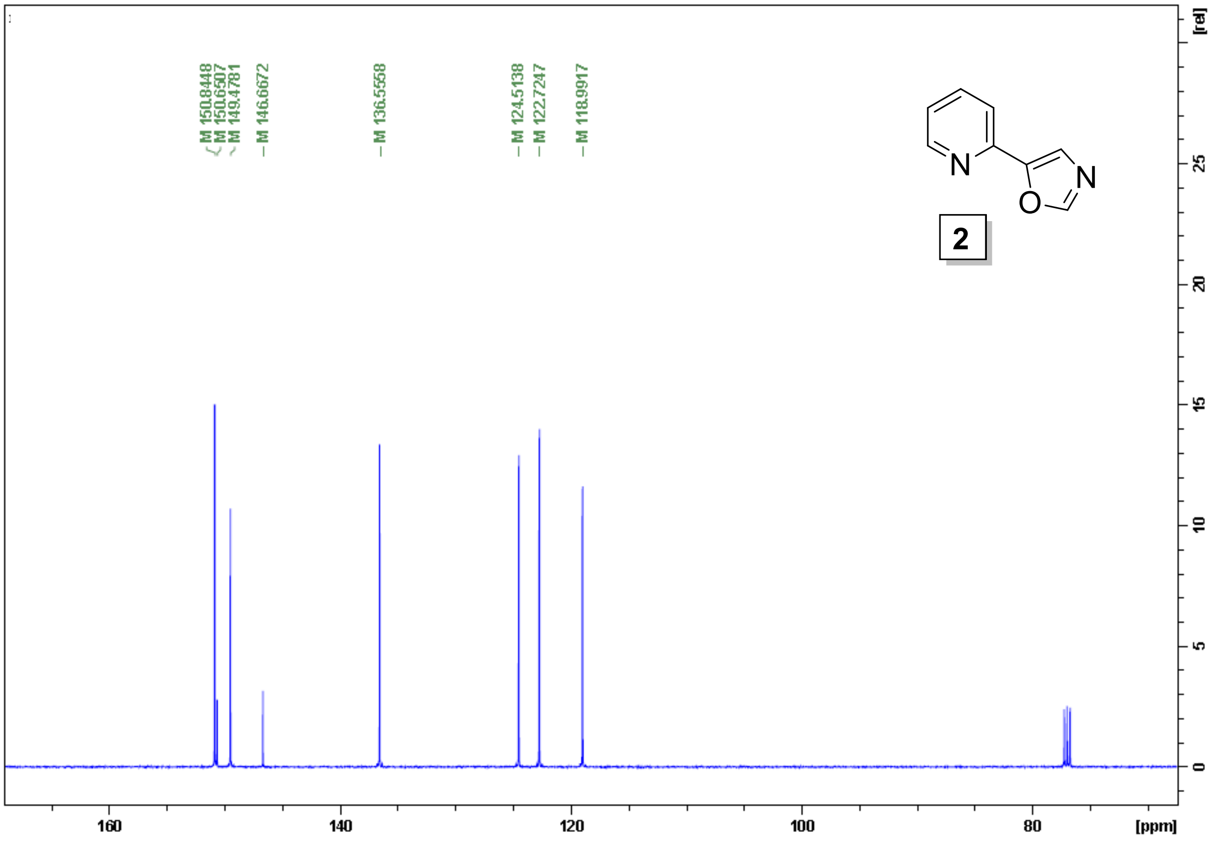
M 150.8448
M 150.6507
M 149.4781
M 146.6672
M 136.5558
M 124.5138
M 122.7247
M 118.9917
2
N
O
N
[rel]
[ppm]

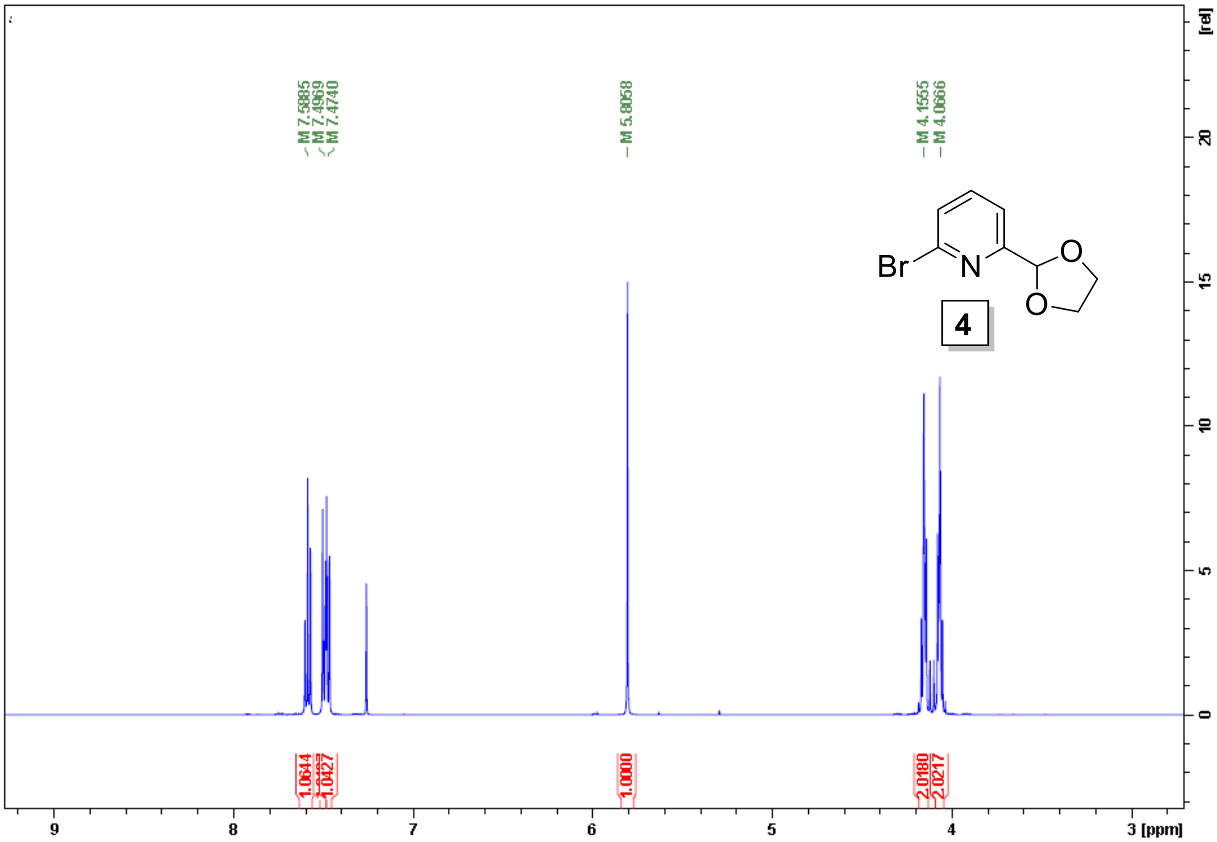
M 7.5885
M 7.4969
M 7.4740
M 5.8058
M 4.1555
M 4.0666
Br
N
O
O
4
1.0644
1.0427
1.0000
2.0180
2.0217
9
8
7
6
5
4
3 [ppm]
[rel]

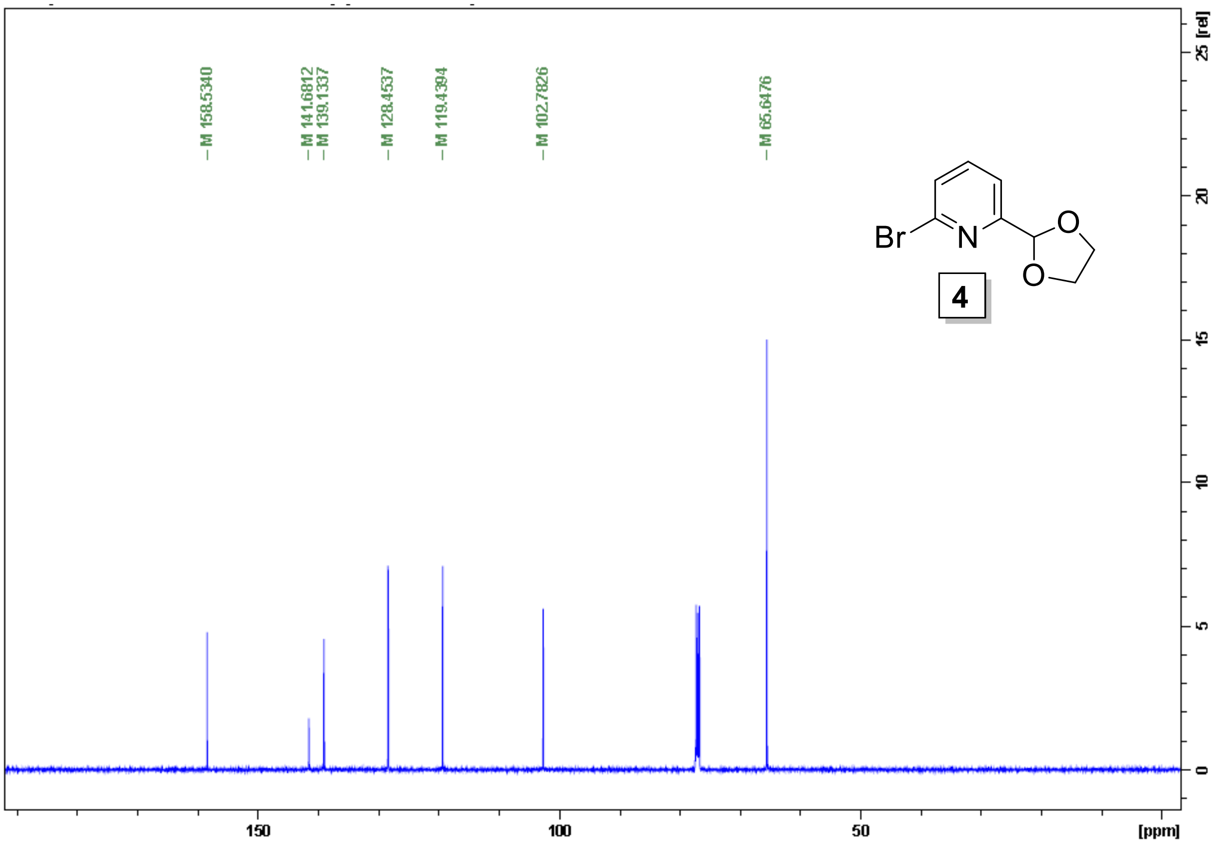
M 158.5340
M 141.6812
M 139.1337
M 128.4537
M 119.4394
M 102.7826
M 65.6476
Br
N
O
O
4
150
100
50
[ppm]
[rel]

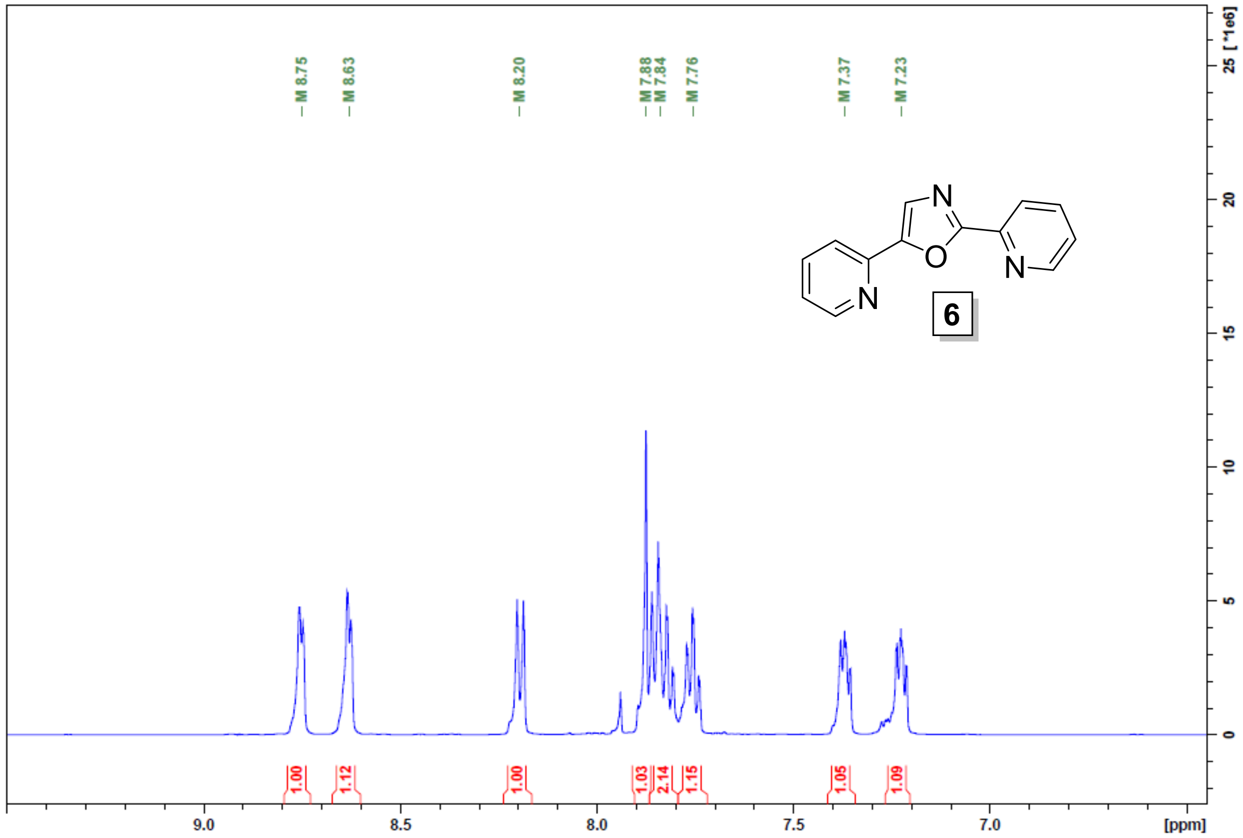
M 8.75
M 8.63
M 8.20
M 7.88
M 7.84
M 7.76
M 7.37
M 7.23
6
1.00
1.12
1.00
1.03
2.14
1.15
1.05
1.09
9.0
8.5
8.0
7.5
7.0
[ppm]
25 [*1e6]

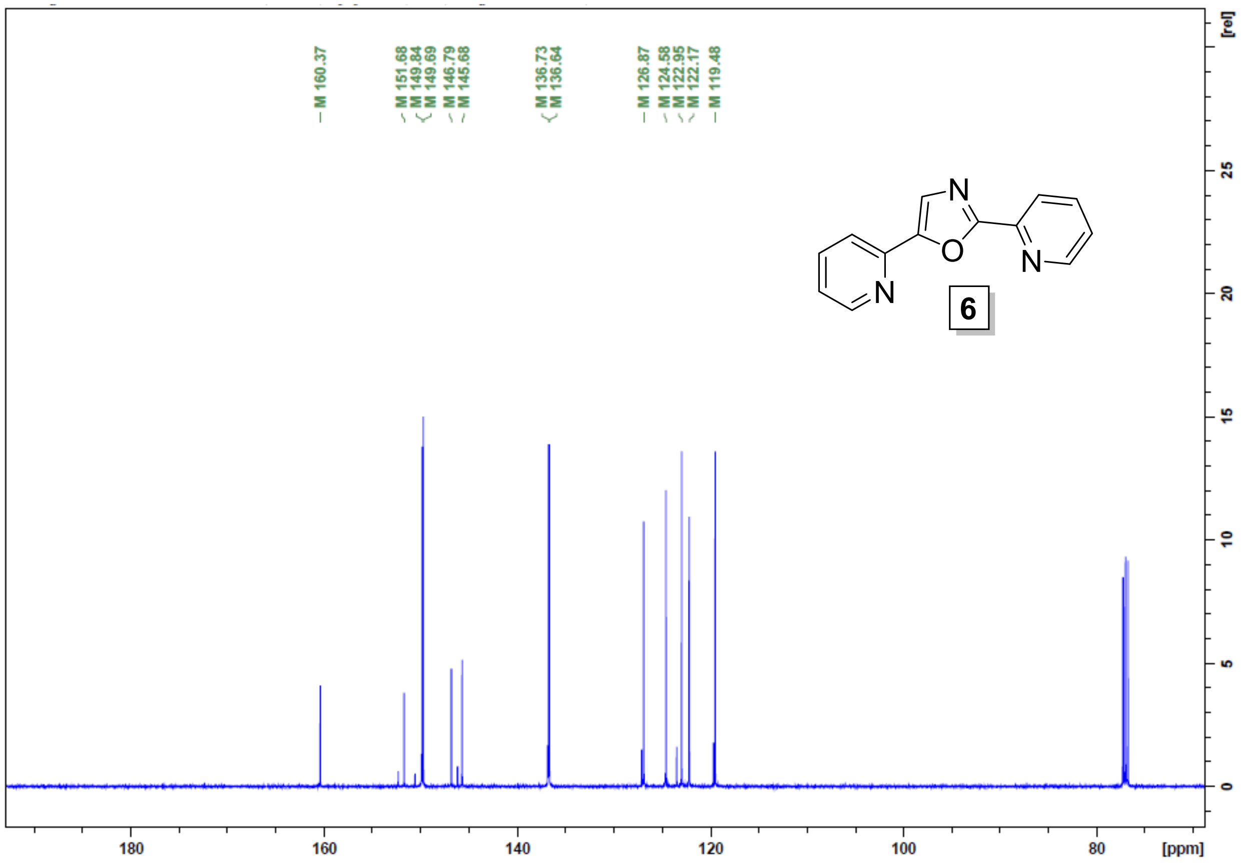
M 160.37
M 151.68
M 149.84
M 149.69
M 146.79
M 145.68
M 136.73
M 136.64
M 126.87
M 124.58
M 122.95
M 122.17
M 119.48
6
180
160
140
120
100
80
[ppm]
[rel]

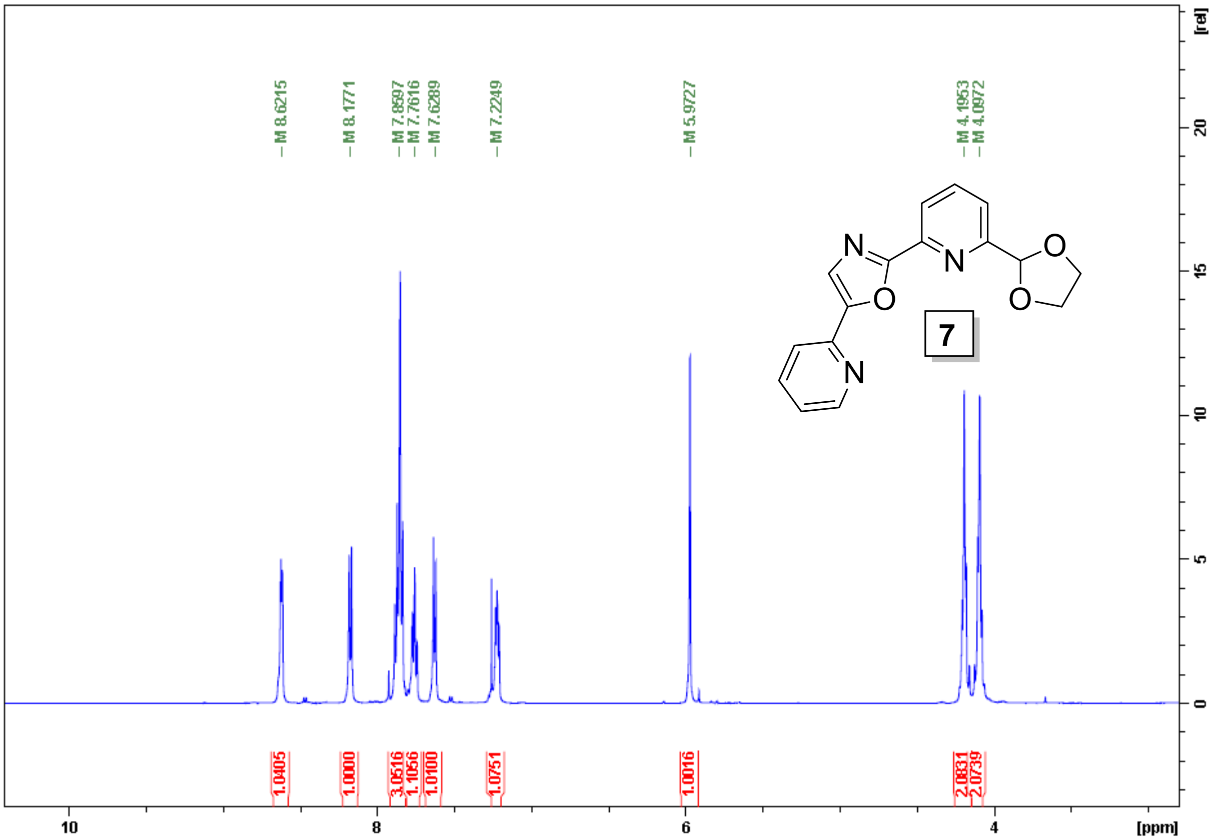
M 8.6215
M 8.1771
M 7.8597
M 7.7616
M 7.6289
M 7.2249
M 5.9727
M 4.1953
M 4.0972
1.0405
1.0000
3.0516
1.1056
1.0100
1.0751
1.0016
2.0831
2.0739
7
[rel]
[ppm]

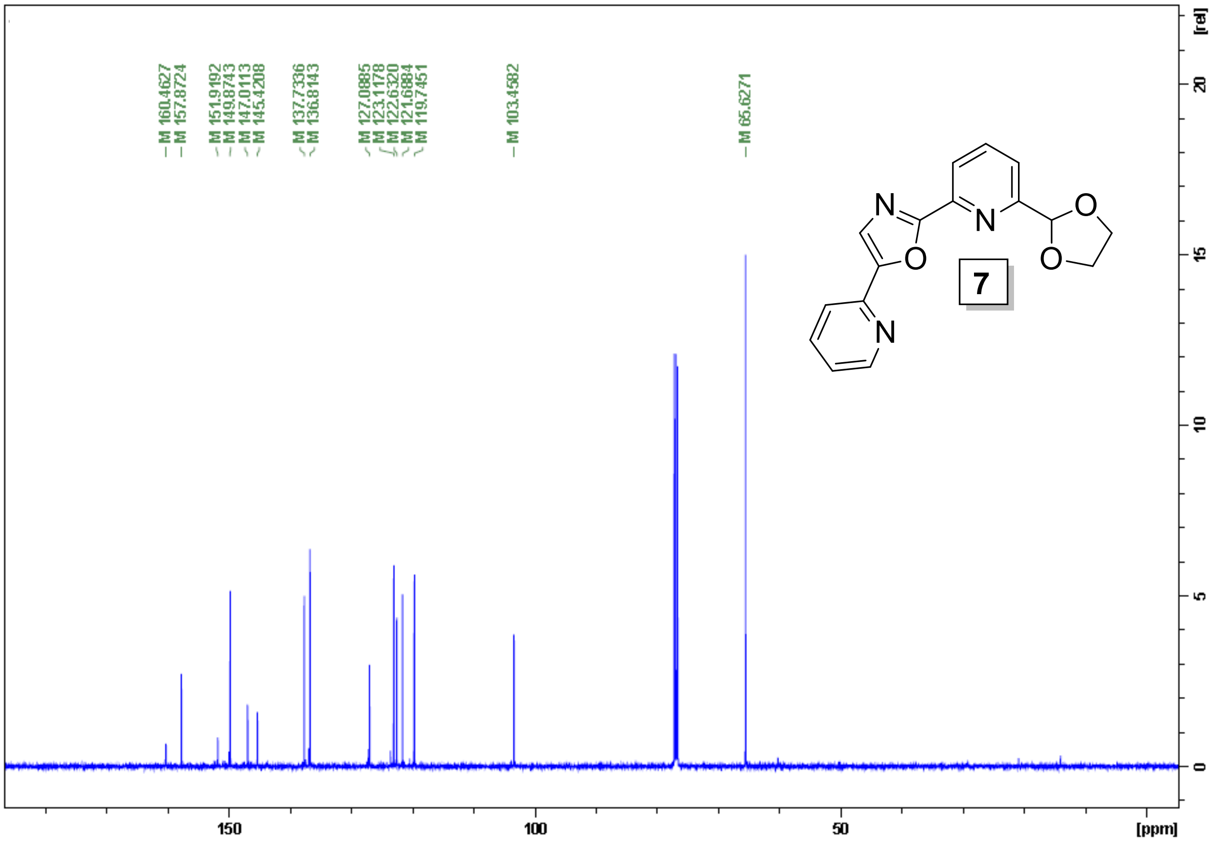
M 160.4627
M 157.8724
M 151.9192
M 149.8743
M 147.0113
M 145.4208
M 137.7336
M 136.8143
M 127.0885
M 123.1178
M 122.6320
M 121.6884
M 119.7451
M 103.4582
M 65.6271
7
[rel]
[ppm]

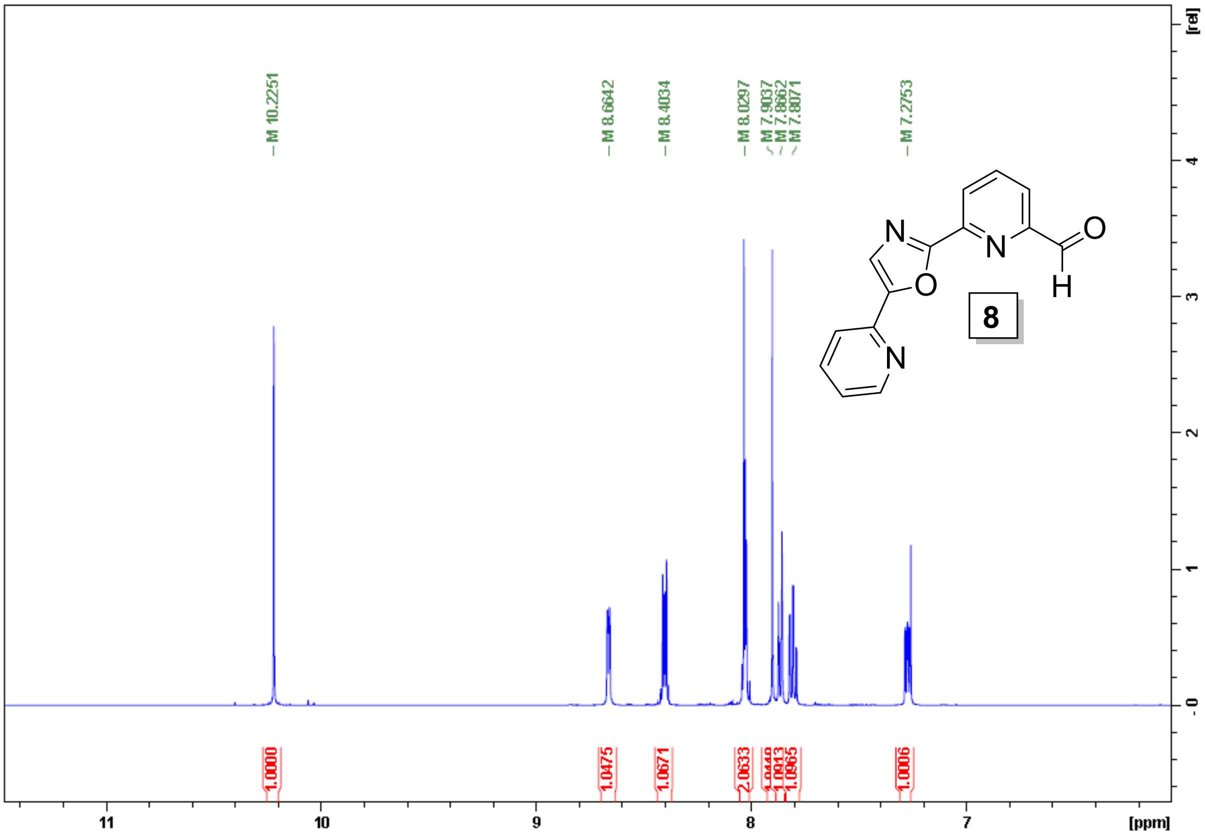

M 10.2251
M 8.6642
M 8.4034
M 8.0297
M 7.9037
M 7.8662
M 7.8071
M 7.2753
1.0000
1.0475
1.0671
2.0633
1.0965
1.0006
8
[rel]
[ppm]

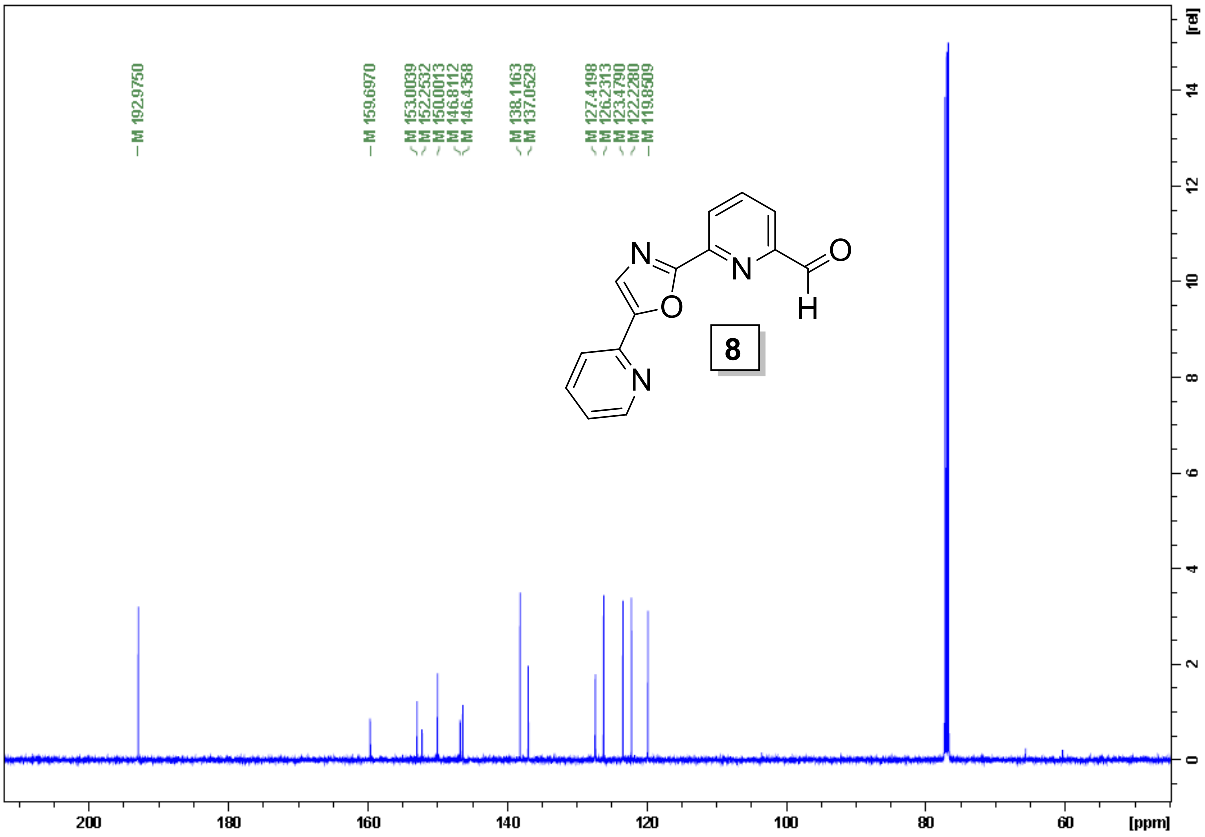

M 192.9750
M 159.6970
M 153.0039
M 152.2532
M 150.0013
M 146.8112
M 146.4358
M 138.1163
M 137.0529
M 127.4198
M 126.2313
M 123.4790
M 122.2280
M 119.8509
8
[rel]
[ppm]

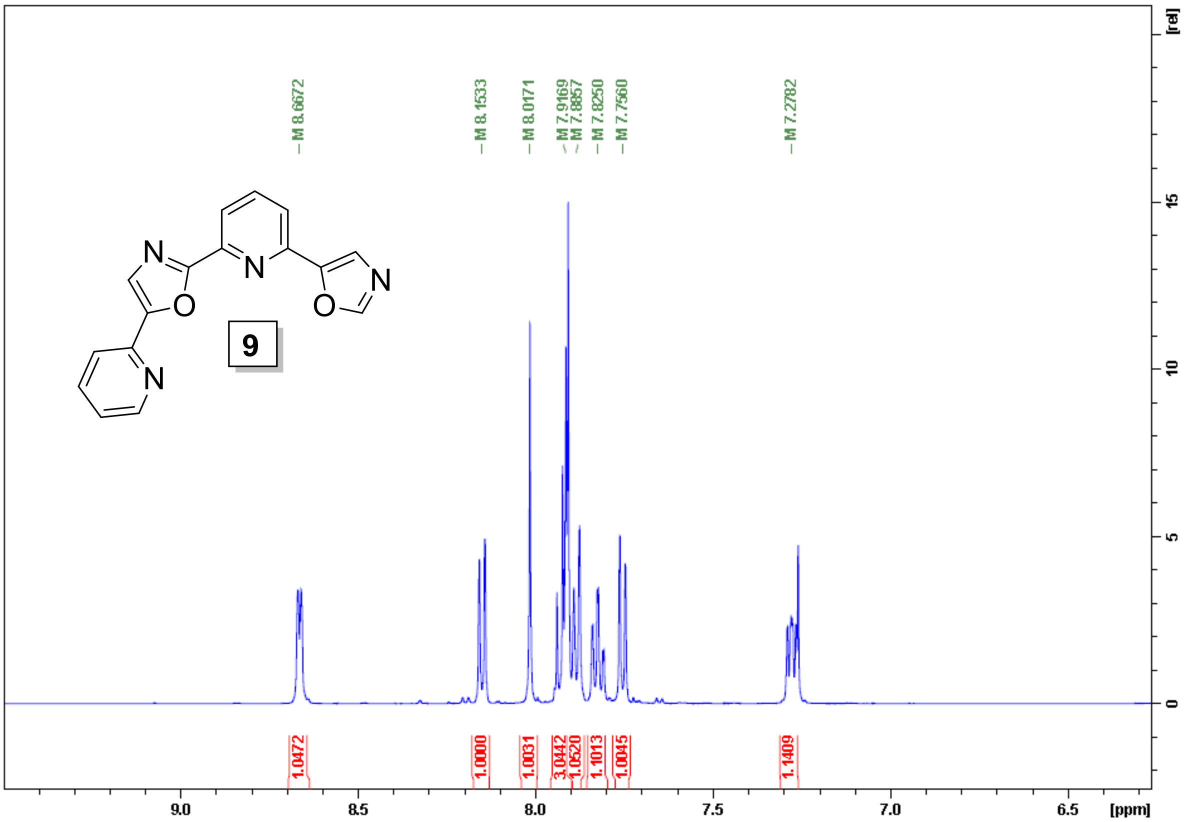
M 8.6672
M 8.1533
M 8.0171
M 7.9169
M 7.8857
M 7.8250
M 7.7560
M 7.2782
1.0472
1.0000
1.0031
3.0442
1.0520
1.1013
1.0045
1.1409
9
[ppm]
[rel]

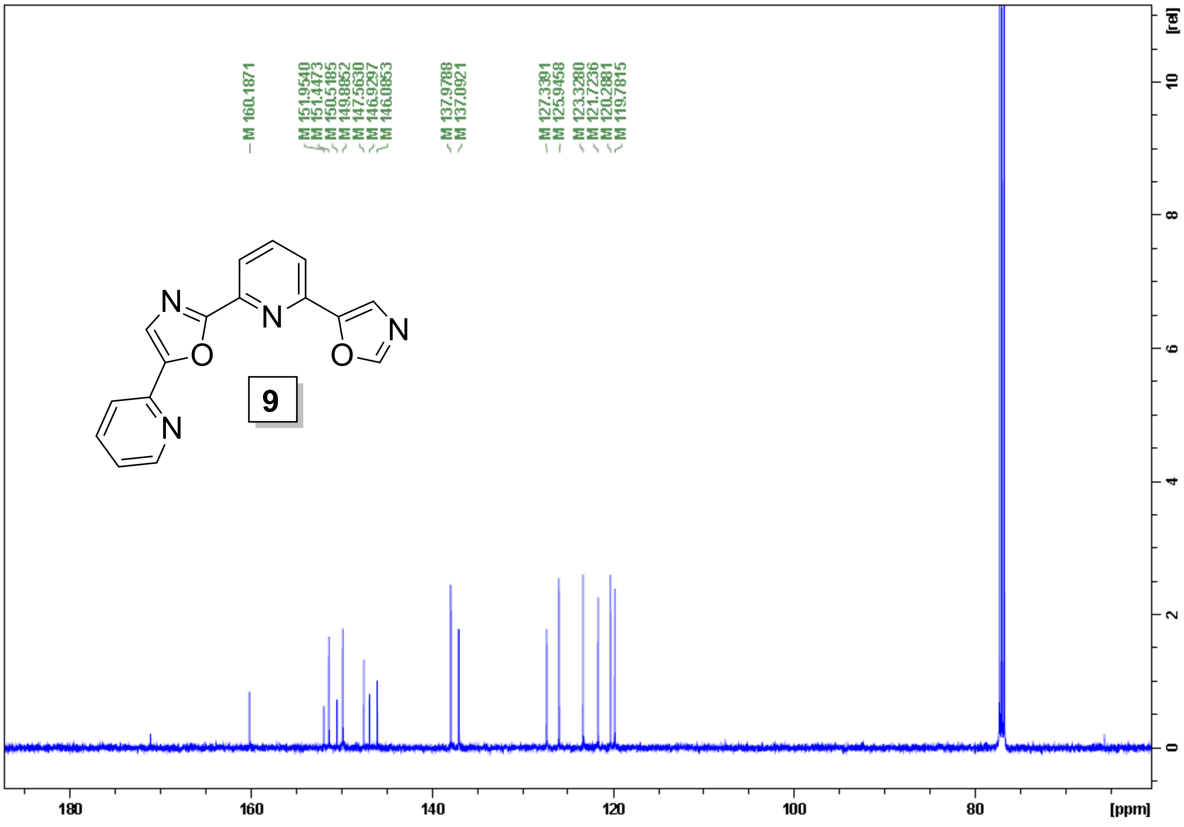
M 160.1871
M 151.9540
M 151.4473
M 150.5185
M 149.8852
M 147.5630
M 146.9297
M 146.0853
M 137.9788
M 137.0921
M 127.3391
M 125.9458
M 123.3280
M 121.7236
M 120.2881
M 119.7815
9
[ppm]
[rel]

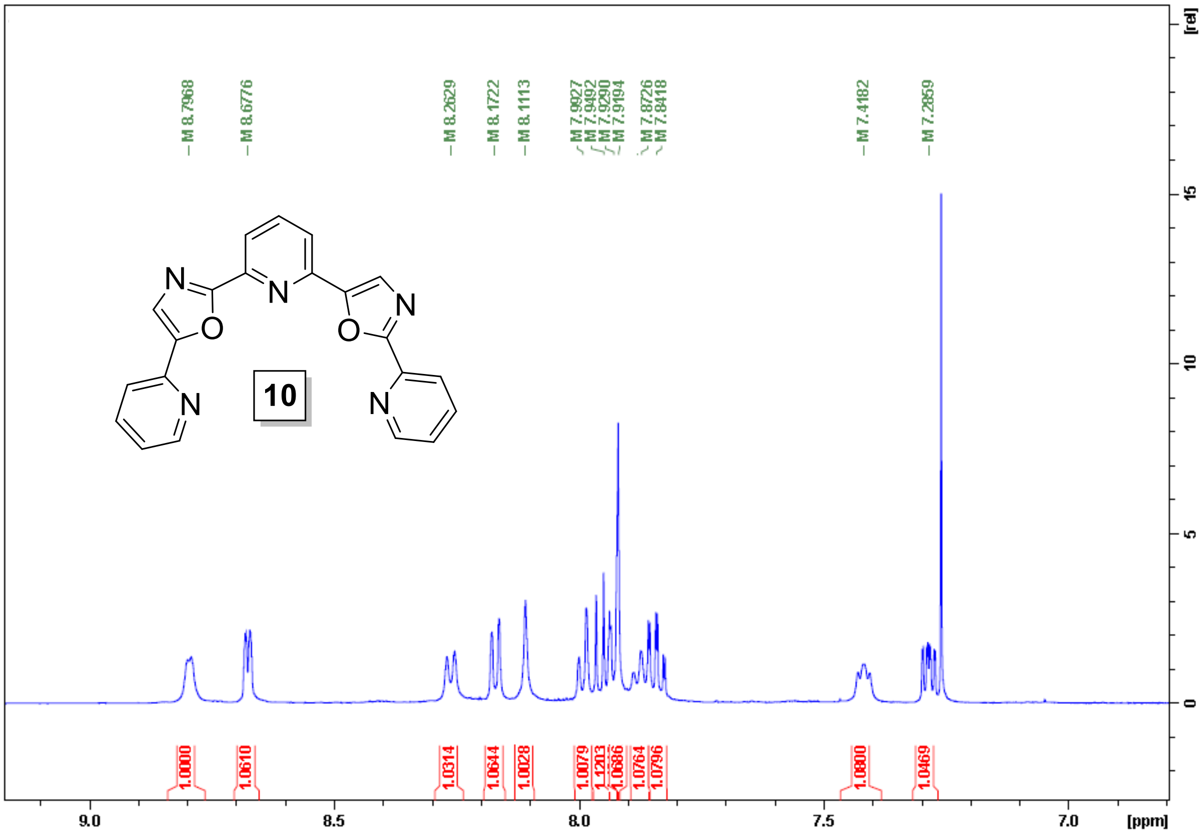
M 8.7968
M 8.6776
M 8.2629
M 8.1722
M 8.1113
M 7.9927
M 7.9492
M 7.9290
M 7.9194
M 7.8726
M 7.8418
M 7.4182
M 7.2859
10
1.0000
1.0610
1.0314
1.0644
1.0028
1.0079
1.1203
1.0686
1.0764
1.0796
1.0800
1.0469
9.0
8.5
8.0
7.5
7.0
[ppm]
[rel]
0
5
10
15

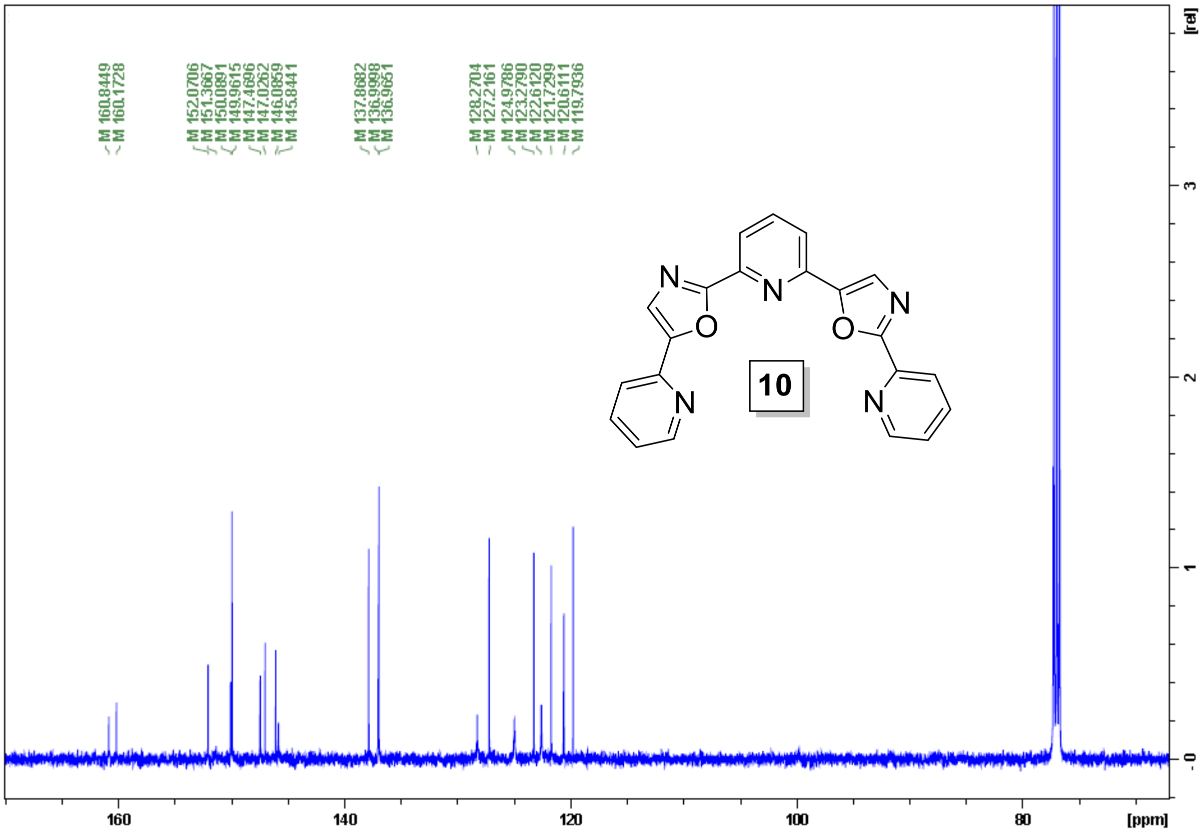
M 160.8449
M 160.1728
M 152.0706
M 151.3667
M 150.0891
M 149.9615
M 147.4696
M 147.0262
M 146.0859
M 145.8441
M 137.8682
M 136.9998
M 136.9651
M 128.2704
M 127.2161
M 124.9786
M 123.2790
M 122.6120
M 121.7299
M 120.6111
M 119.7936
10
160
140
120
100
80
[ppm]
[rel]
0
1
2
3

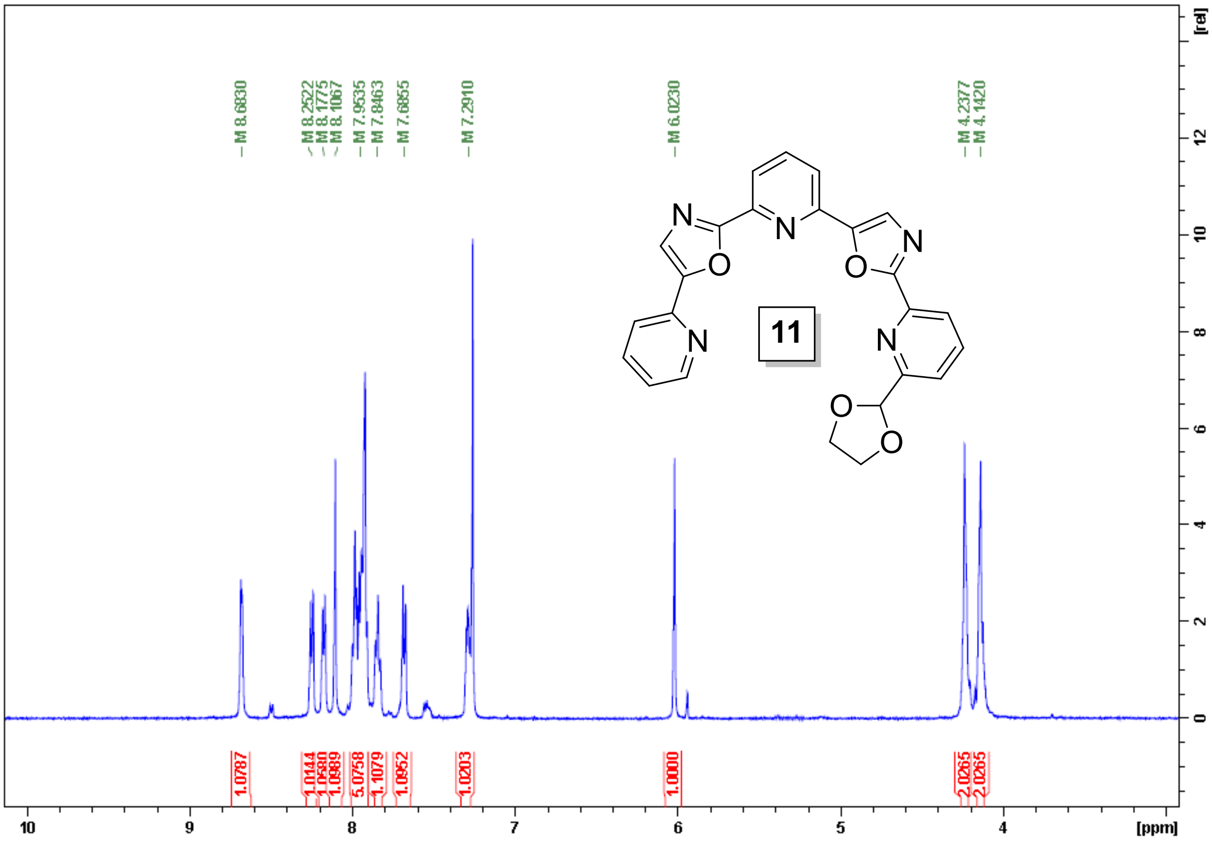
M 8.6830
M 8.2522
M 8.1775
M 8.1067
M 7.9535
M 7.8463
M 7.6855
M 7.2910
M 6.0230
M 4.2377
M 4.1420
11
1.0787
1.0144
1.0580
1.0989
5.0758
1.1079
1.0952
1.0203
1.0000
2.0265
2.0265
[rel]
[ppm]

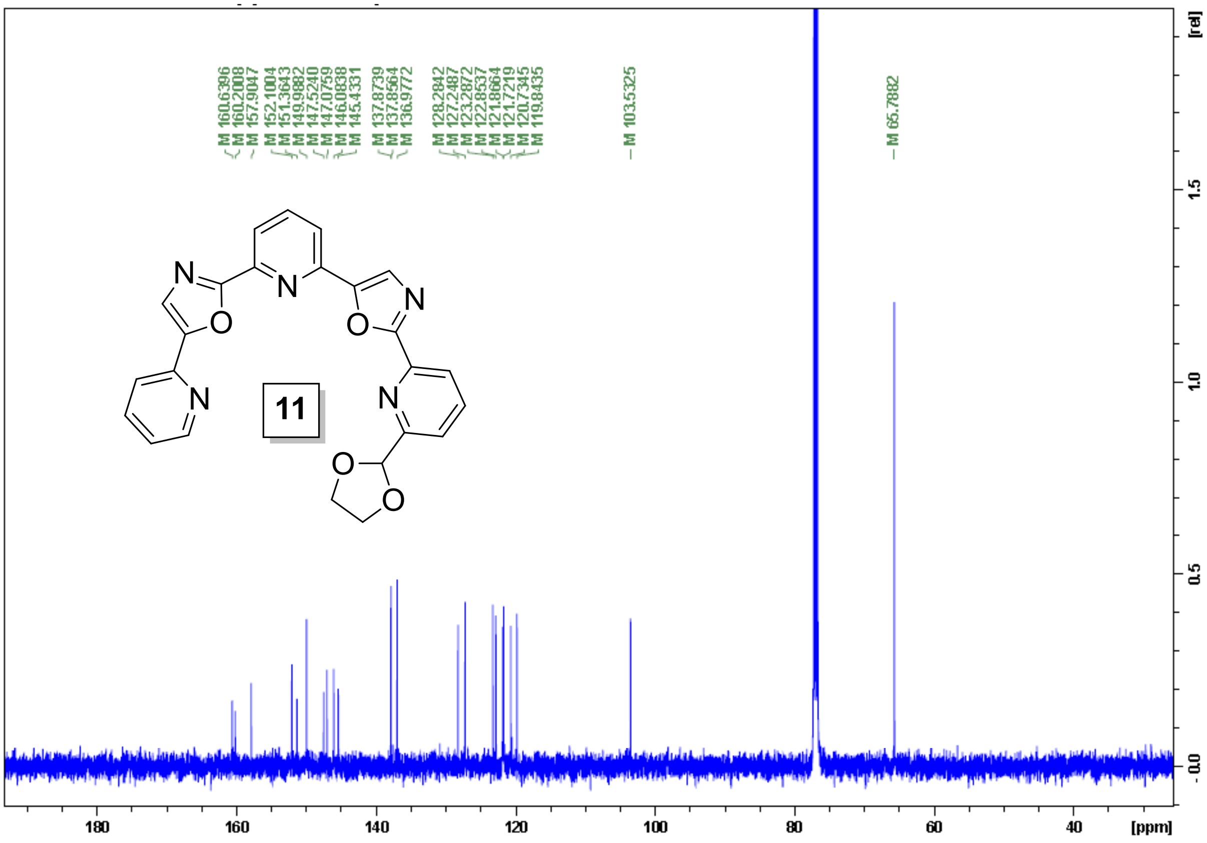
M 160.6396
M 160.2008
M 157.9047
M 152.1004
M 151.3643
M 149.9882
M 147.5240
M 147.0759
M 146.0838
M 145.4331
M 137.8739
M 137.8564
M 136.9772
M 128.2842
M 127.2487
M 123.2872
M 122.8537
M 121.8664
M 121.7219
M 120.7345
M 119.8435
M 103.5325
M 65.7882
11
[rel]
[ppm]

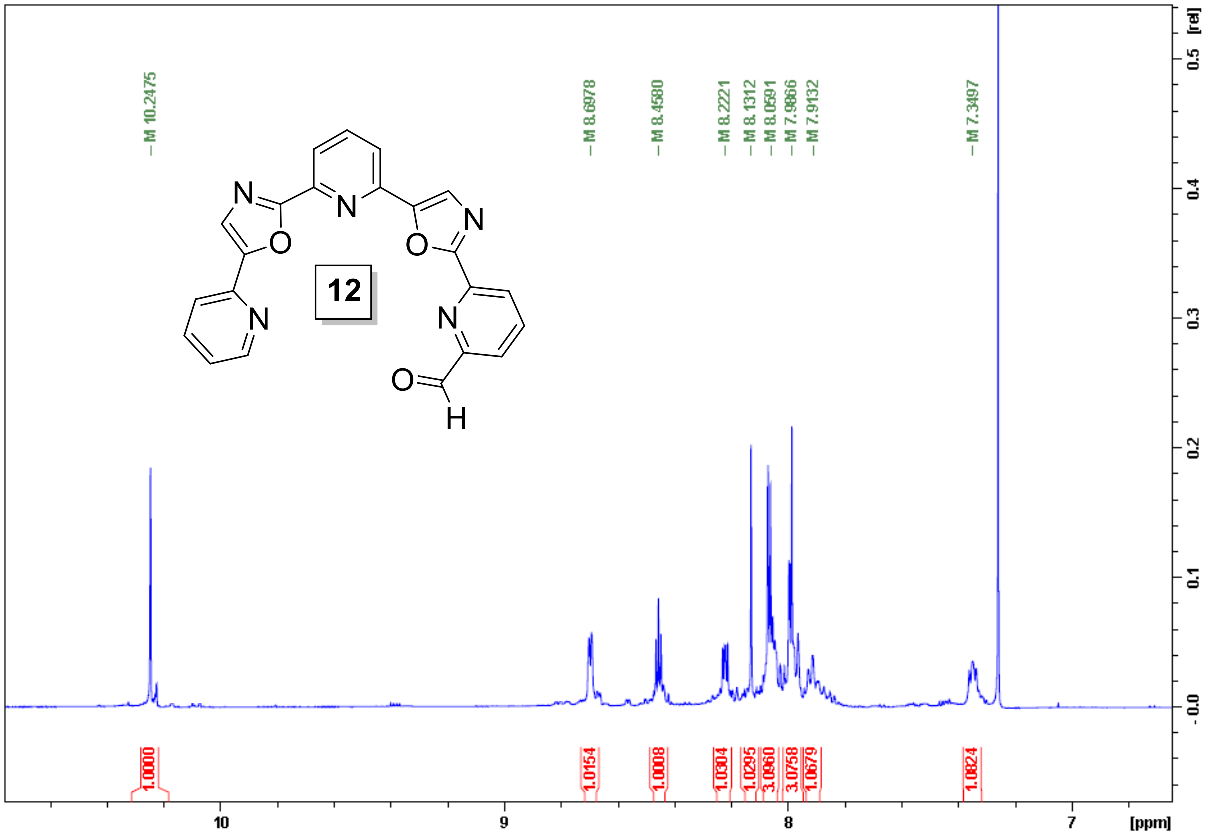
M 10.2475
M 8.6978
M 8.4580
M 8.2221
M 8.1312
M 8.0591
M 7.9866
M 7.9132
M 7.3497
12
1.0000
1.0154
1.0008
1.0304
1.0295
3.0960
3.0758
1.0679
1.0824
10
9
8
7
[ppm]
[rel]

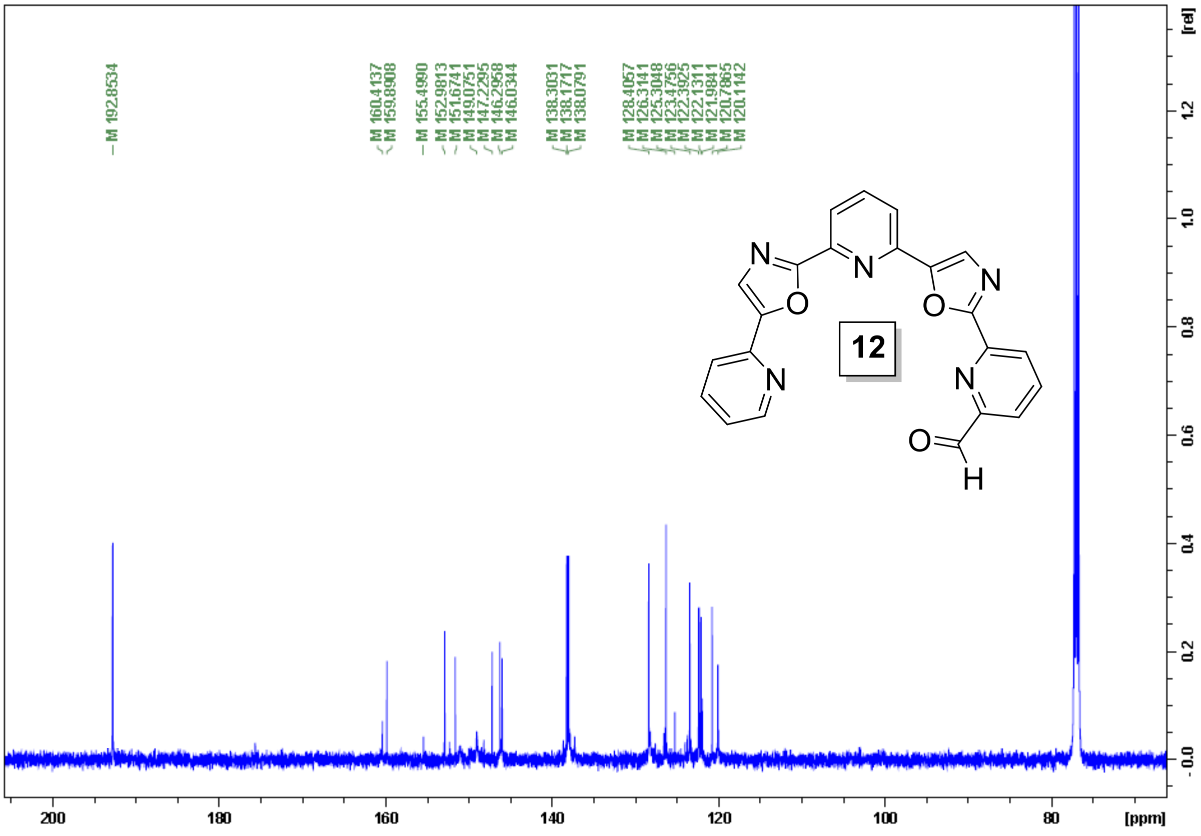
M 192.8534
M 160.4137
M 159.8908
M 155.4990
M 152.9813
M 151.6741
M 149.0751
M 147.2295
M 146.2958
M 146.0344
M 138.3031
M 138.1717
M 138.0791
M 128.4057
M 126.3141
M 125.3048
M 123.4756
M 122.3925
M 122.1311
M 121.9841
M 120.7865
M 120.1142
12
200
180
160
140
120
100
80
[ppm]
[rel]

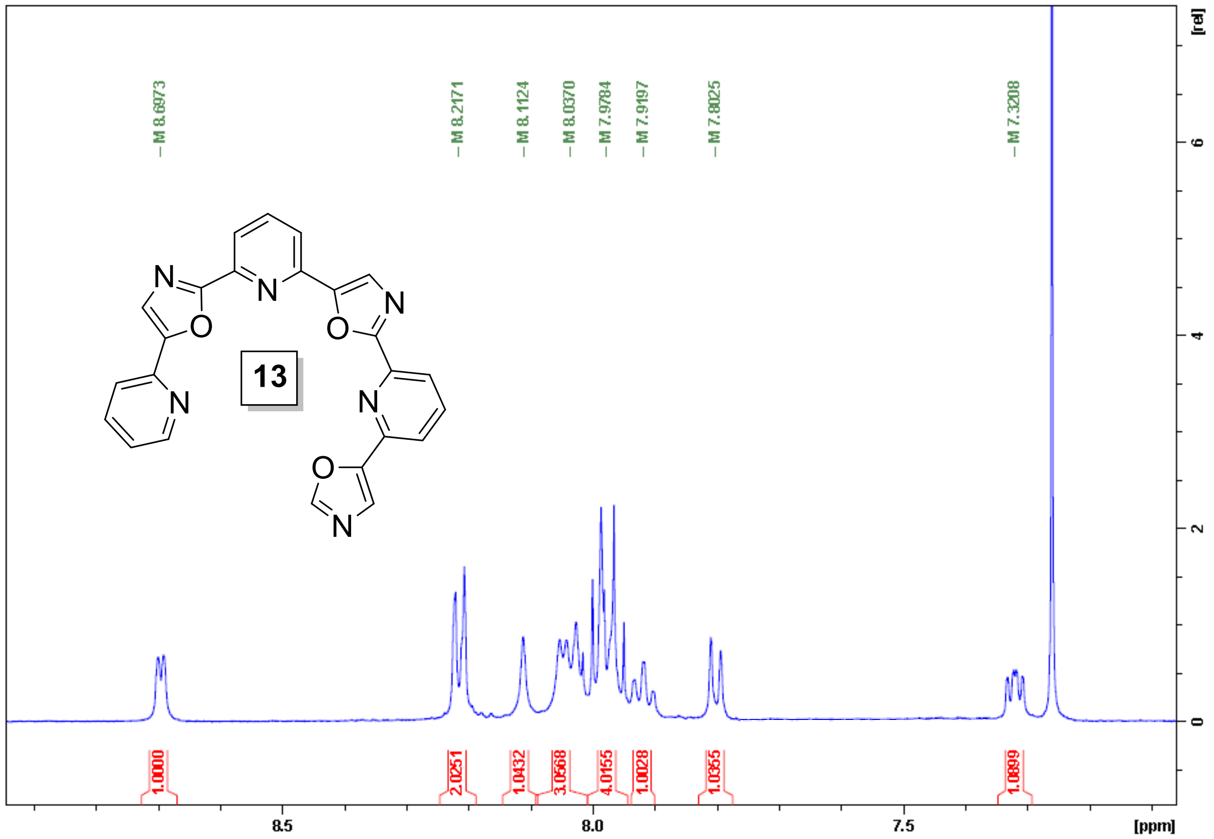
M 8.6973
M 8.2171
M 8.1124
M 8.0370
M 7.9784
M 7.9197
M 7.8025
M 7.3208
1.0000
2.0251
1.0432
3.0568
4.0155
1.0028
1.0355
1.0899
13
[rel]
[ppm]
8.5
8.0
7.5

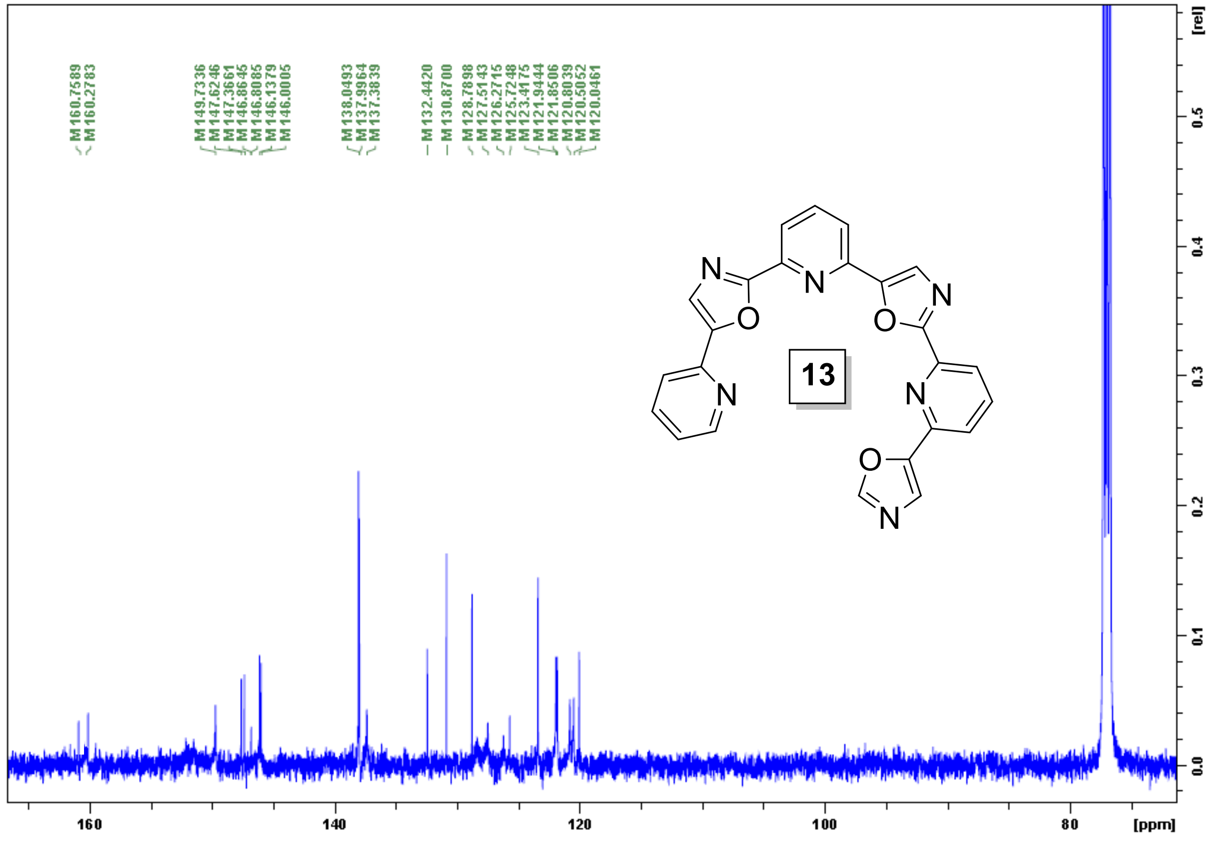
M 160.7589
M 160.2783
M 149.7336
M 147.6246
M 147.3661
M 146.8645
M 146.8085
M 146.1379
M 146.0005
M 138.0493
M 137.9964
M 137.3839
M 132.4420
M 130.8700
M 128.7898
M 127.5143
M 126.2715
M 125.7248
M 123.4175
M 121.9444
M 121.8506
M 120.8039
M 120.5052
M 120.0461
13
[rel]
[ppm]
160
140
120
100
80

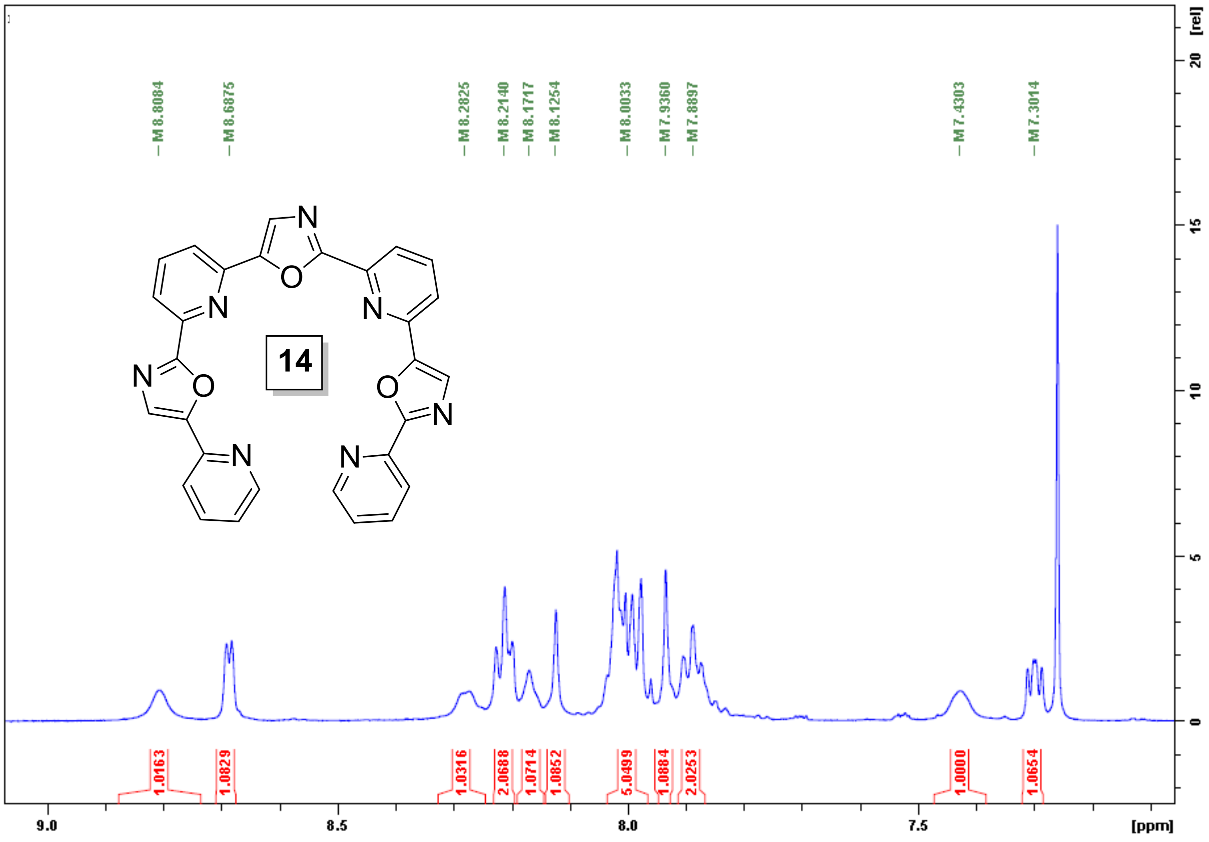
M 8.8084
M 8.6875
M 8.2825
M 8.2140
M 8.1717
M 8.1254
M 8.0033
M 7.9360
M 7.8897
M 7.4303
M 7.3014
1.0163
1.0829
1.0316
2.0688
1.0714
1.0852
5.0499
1.0884
2.0253
1.0000
1.0654
14
9.0
8.5
8.0
7.5
[ppm]
[rel]

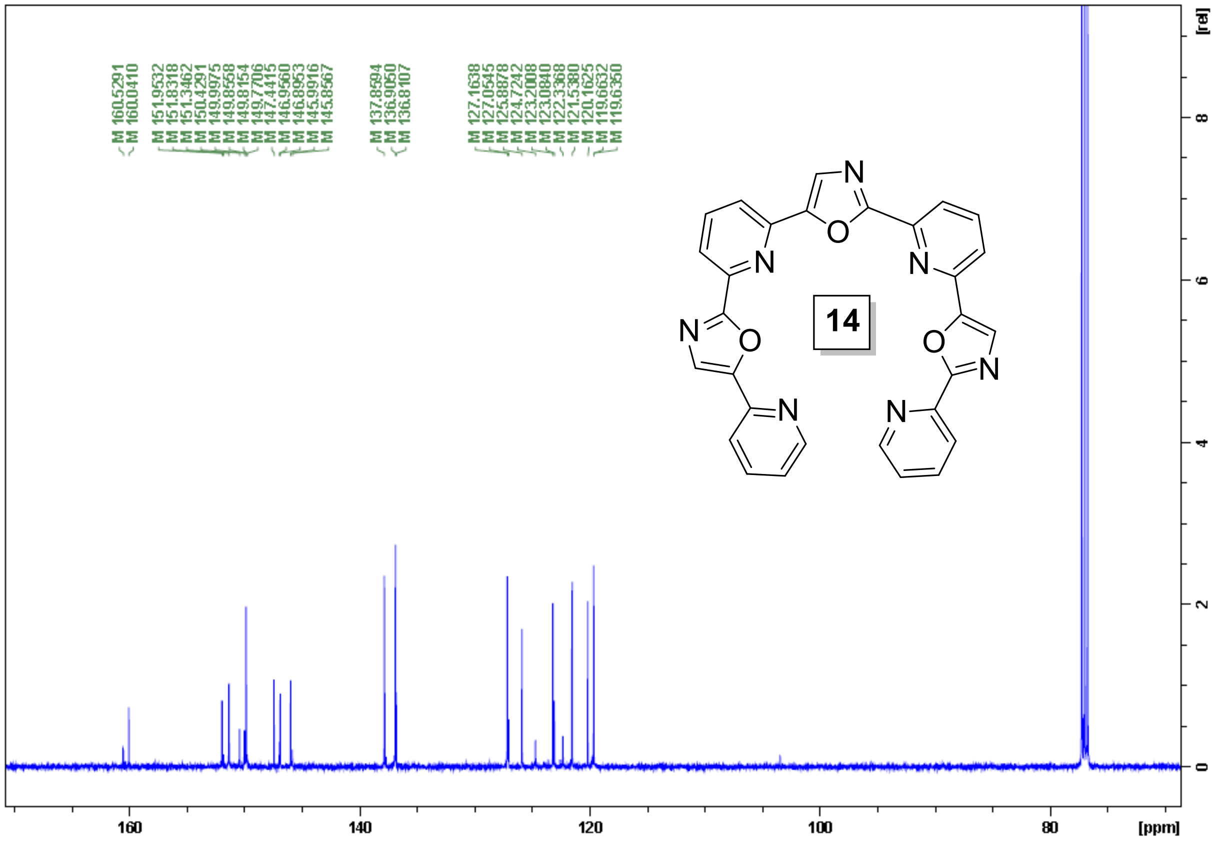
M 160.5291
M 160.0410
M 151.9532
M 151.8318
M 151.3462
M 150.4291
M 149.9975
M 149.8558
M 149.8154
M 149.7706
M 147.4415
M 146.9560
M 146.8953
M 145.9916
M 145.8567
M 137.8594
M 136.9050
M 136.8107
M 127.1638
M 127.0545
M 125.8878
M 124.7242
M 123.2008
M 123.0840
M 122.3368
M 121.5380
M 120.1625
M 119.6632
M 119.6350
14
160
140
120
100
80
[ppm]
[rel]

22AG (telomeric)/K^{+}/5-mer (**10**):

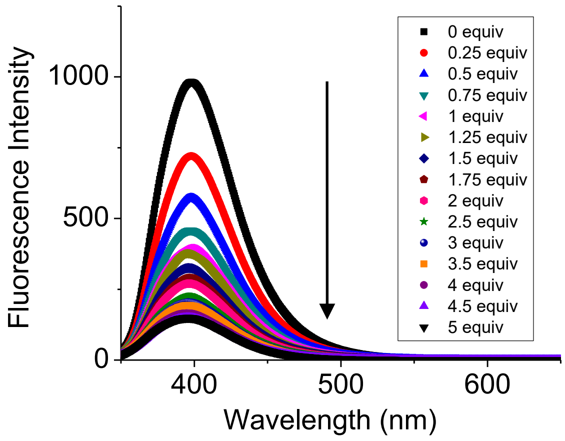

22AG (telomeric)/K^{+}/6-mer (**13**):

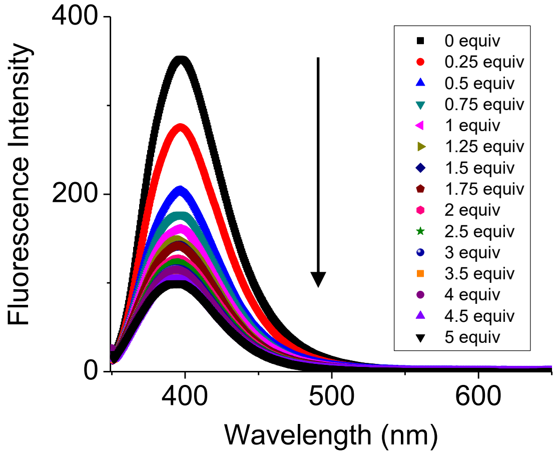

22AG (telomeric)/K^{+}/7-mer (**14**):

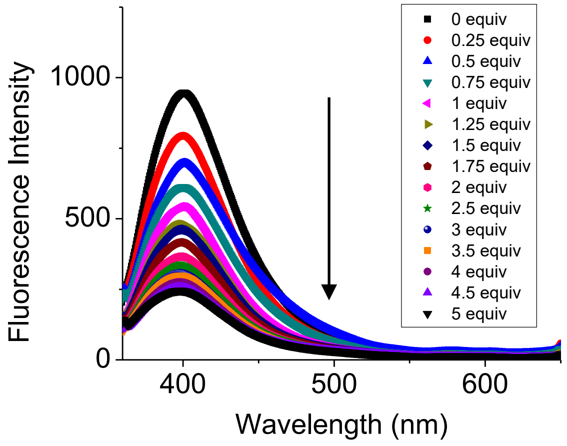

22AG (telomeric)/Na^+/5-mer (**10**):

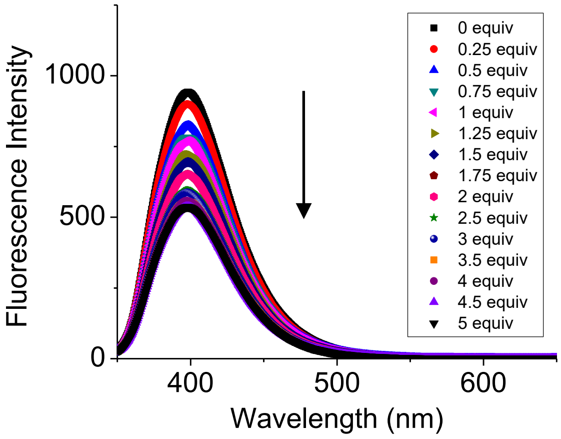

22AG (telomeric)/Na^+/6-mer (**13**):

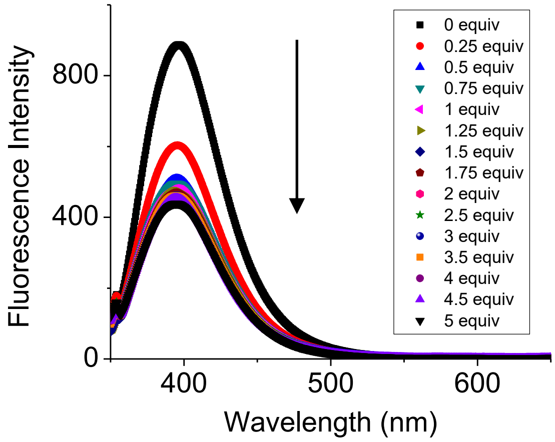

22AG (telomeric)/Na^+/7-mer (**14**):

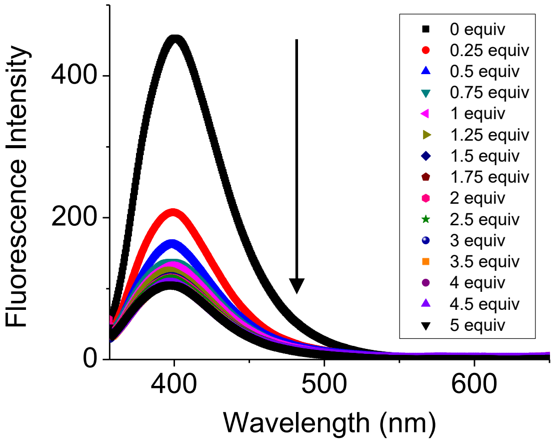

Myc2345-Pu22/K^+/5-mer (**10**):

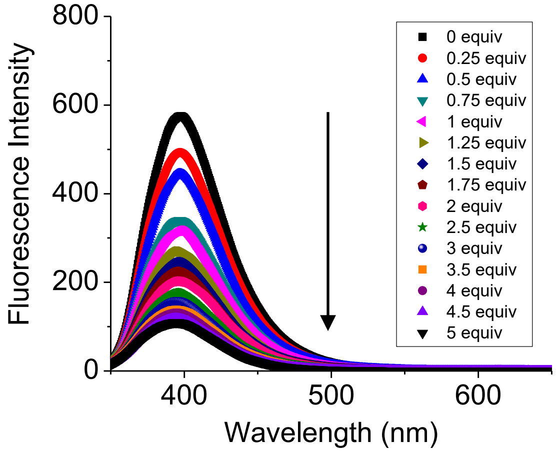

Myc2345-Pu22/K^+/6-mer (**13**):

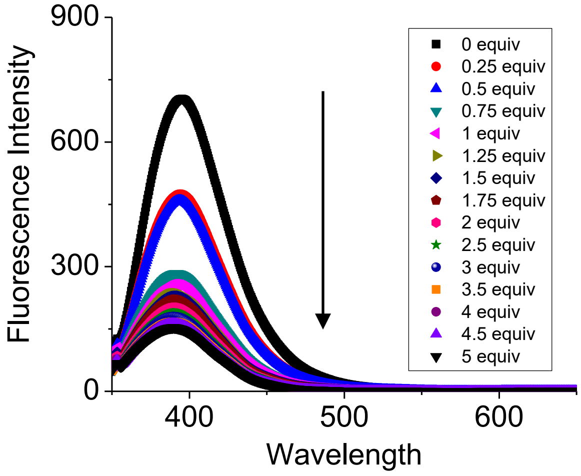

Myc2345-Pu22/K^+/7-mer (**14**):

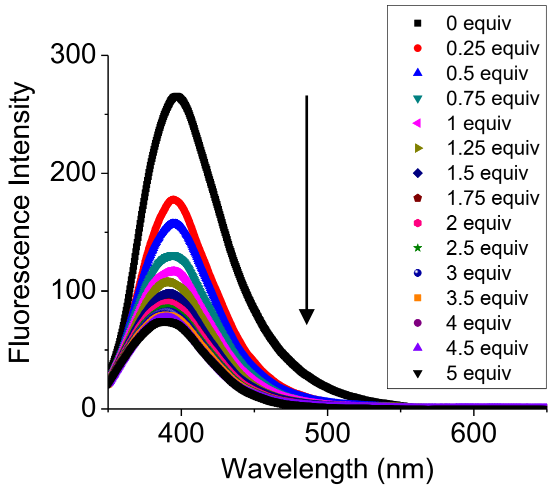

F21T/K$^+$/7mer (**14**)/ds26:

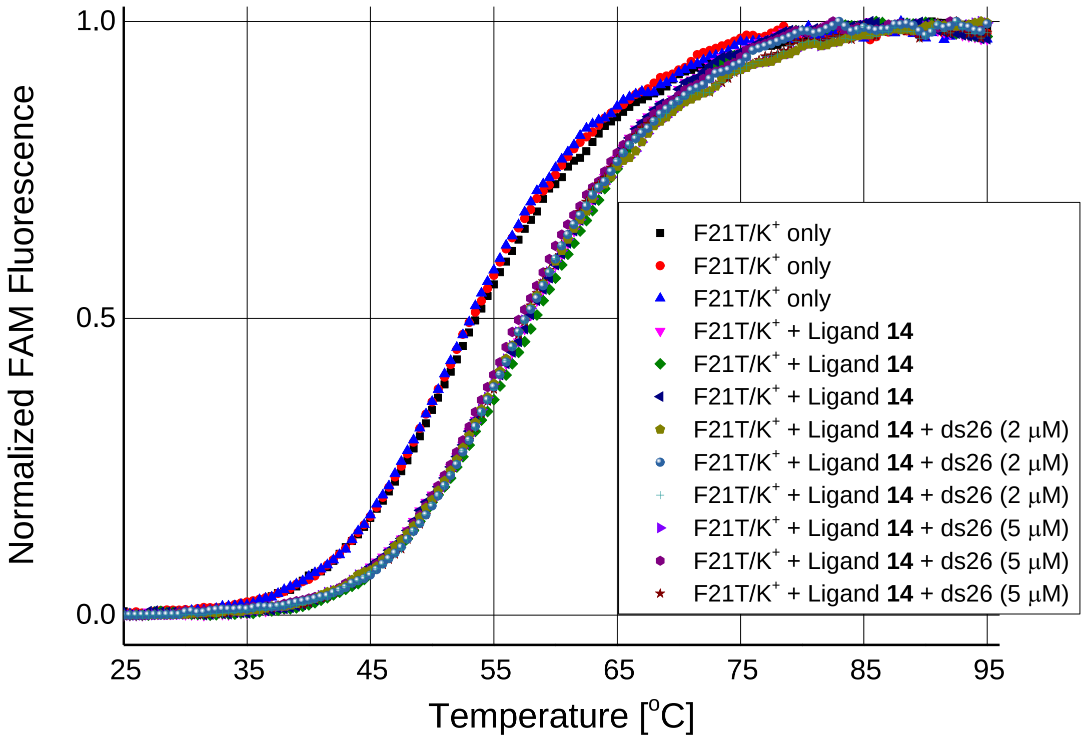

F21T/K$^+$/6mer (**13**)/ds26:

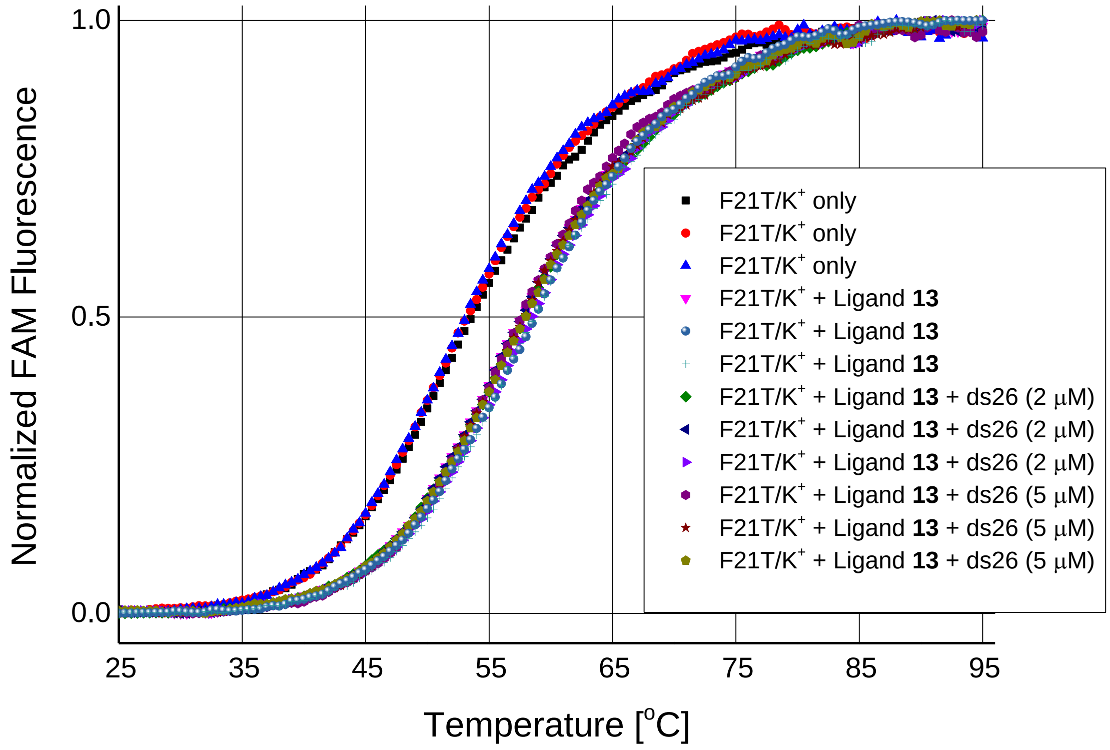

F21T/K$^+$/5mer (**10**)/ds26:

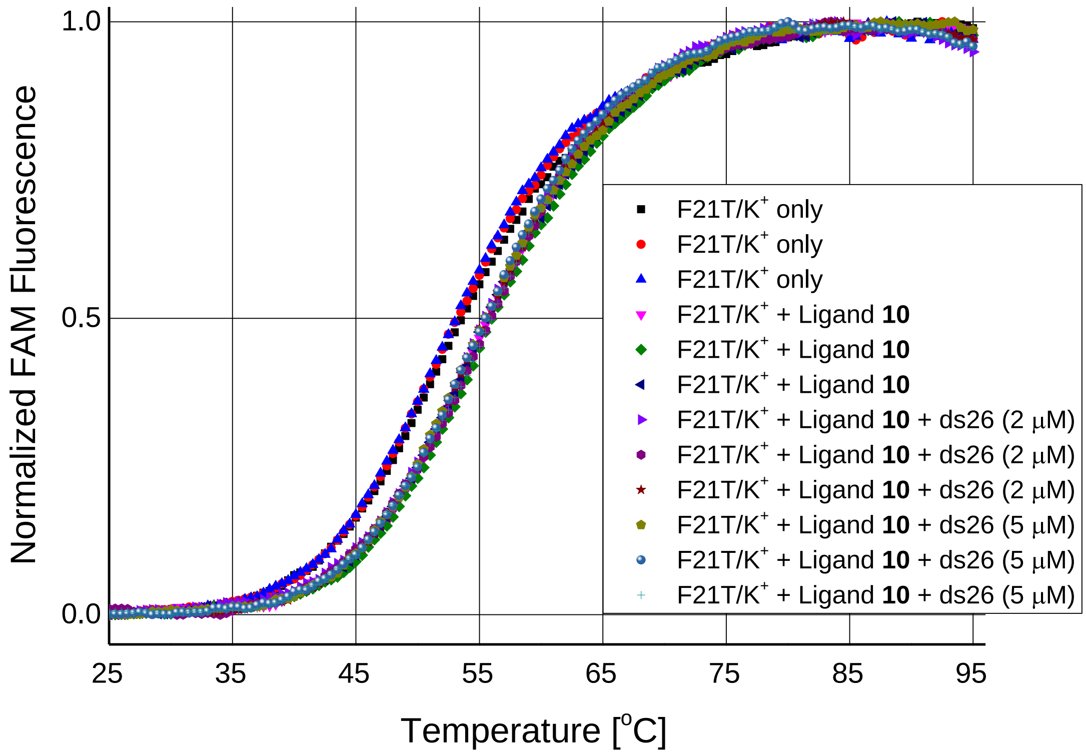

F21T/Na$^+$/7mer (**14**)/ds26:

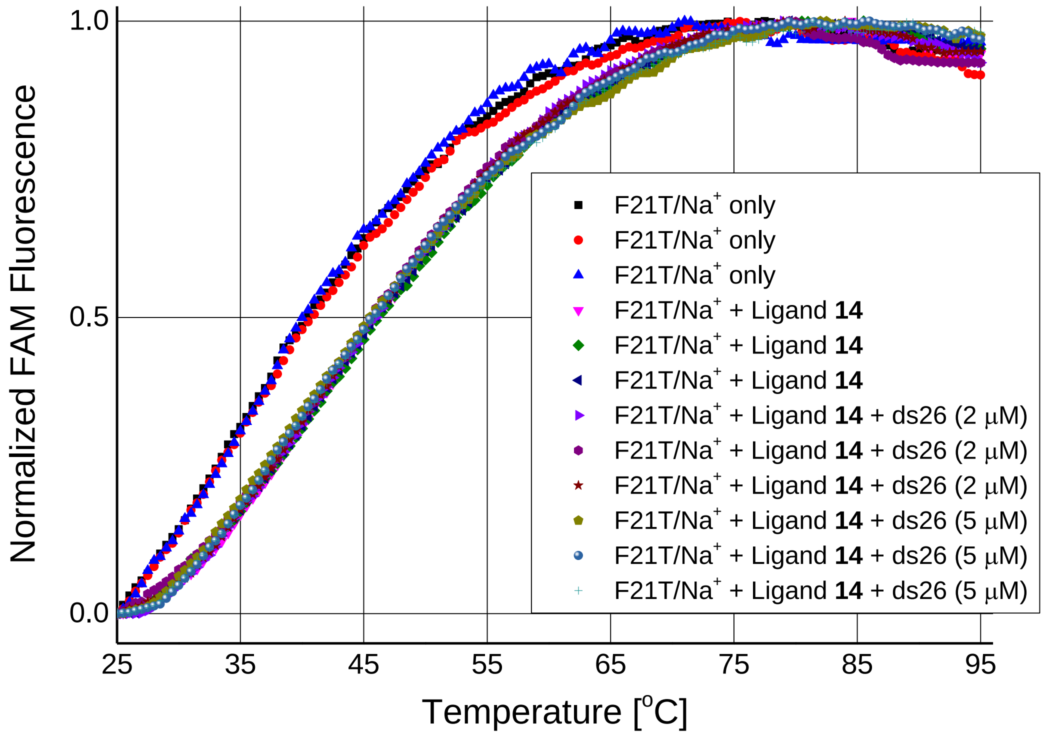

F21T/Na^+/6mer (**13**)/ds26:

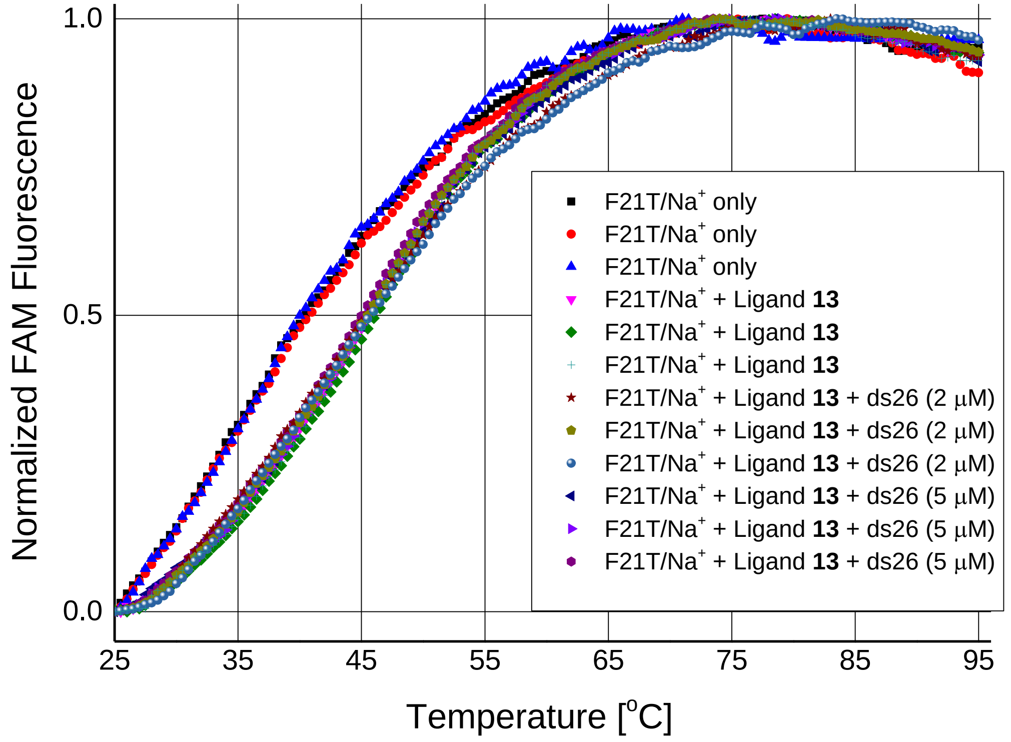

F21T/Na^+/5mer (**10**)/ds26:

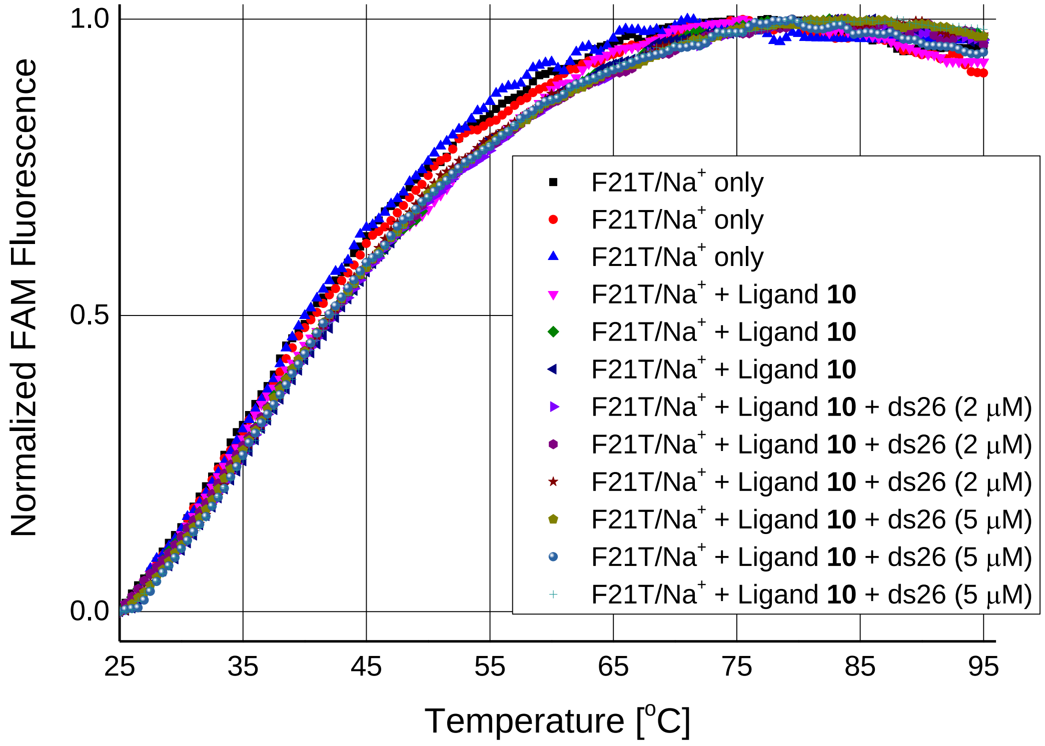

FmycT/K$^+$/7mer (**14**)/ds26:

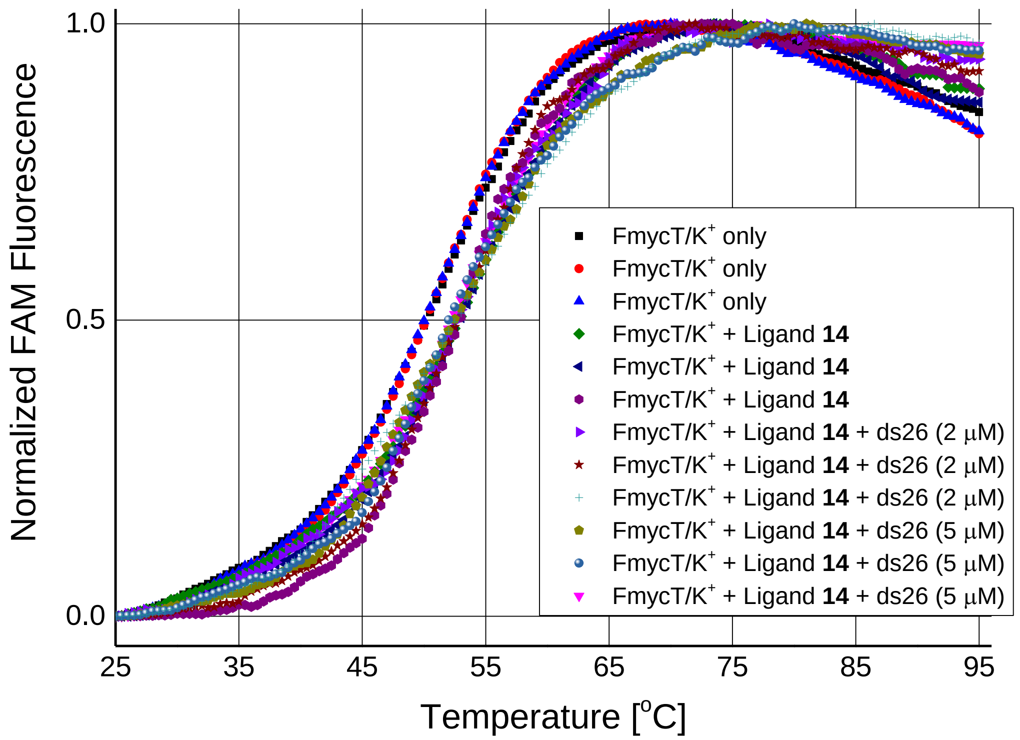

FmycT/K$^+$/6mer (**13**)/ds26:

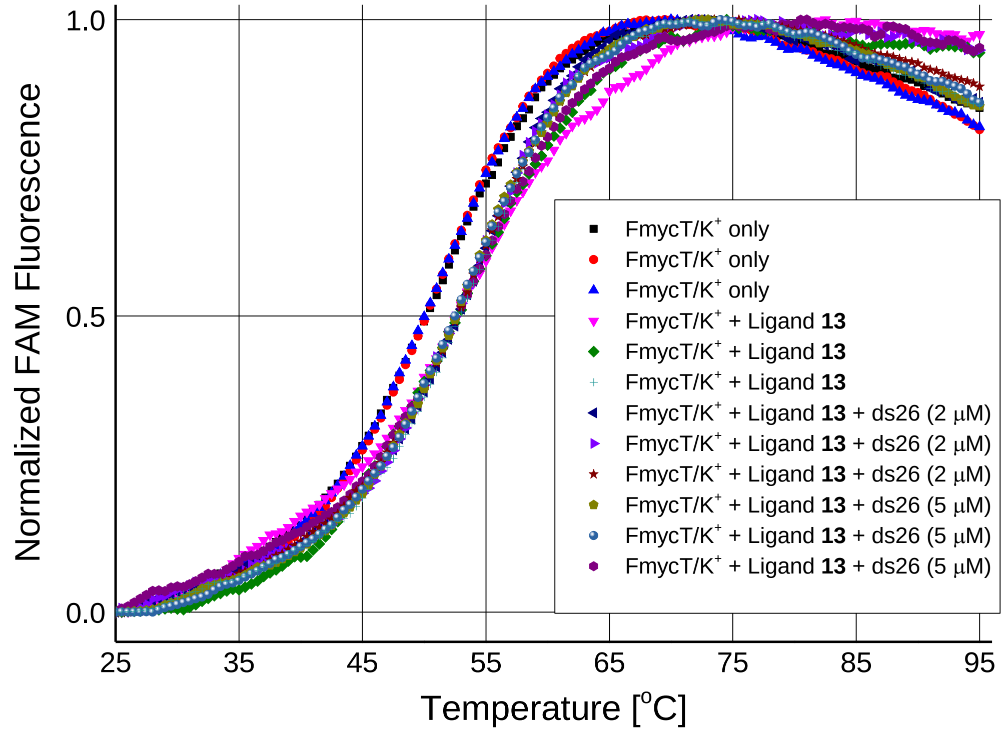

FmycT/K^+/5mer (**10**)/ds26:

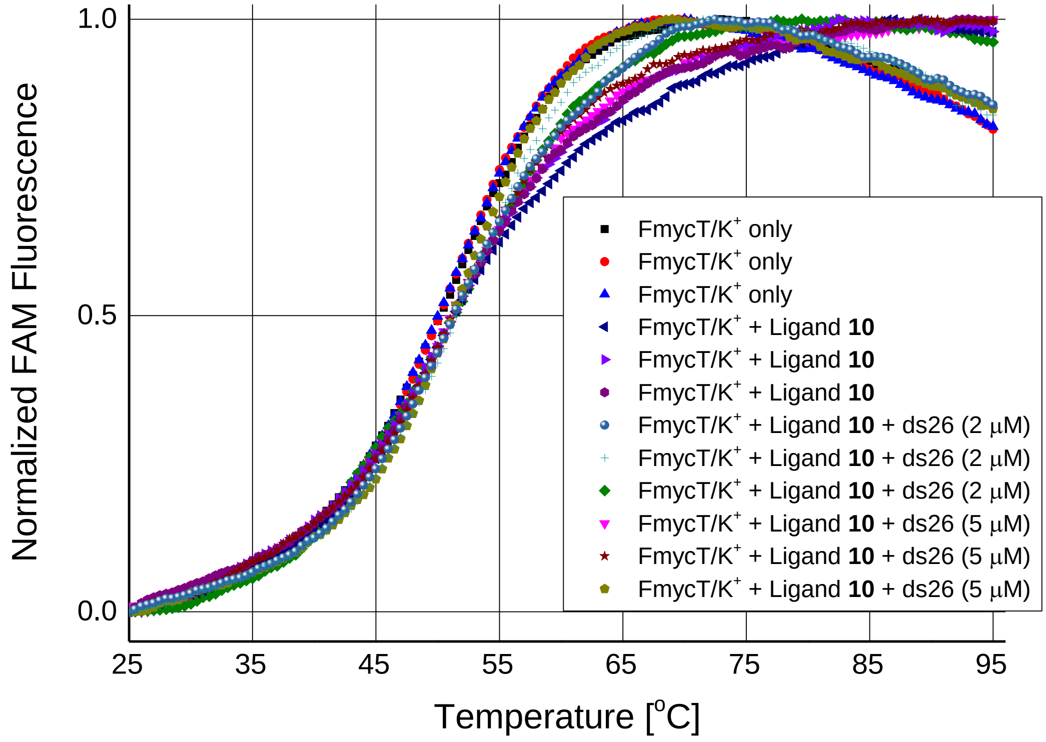

Fds26T/K^+/7mer (**14**):

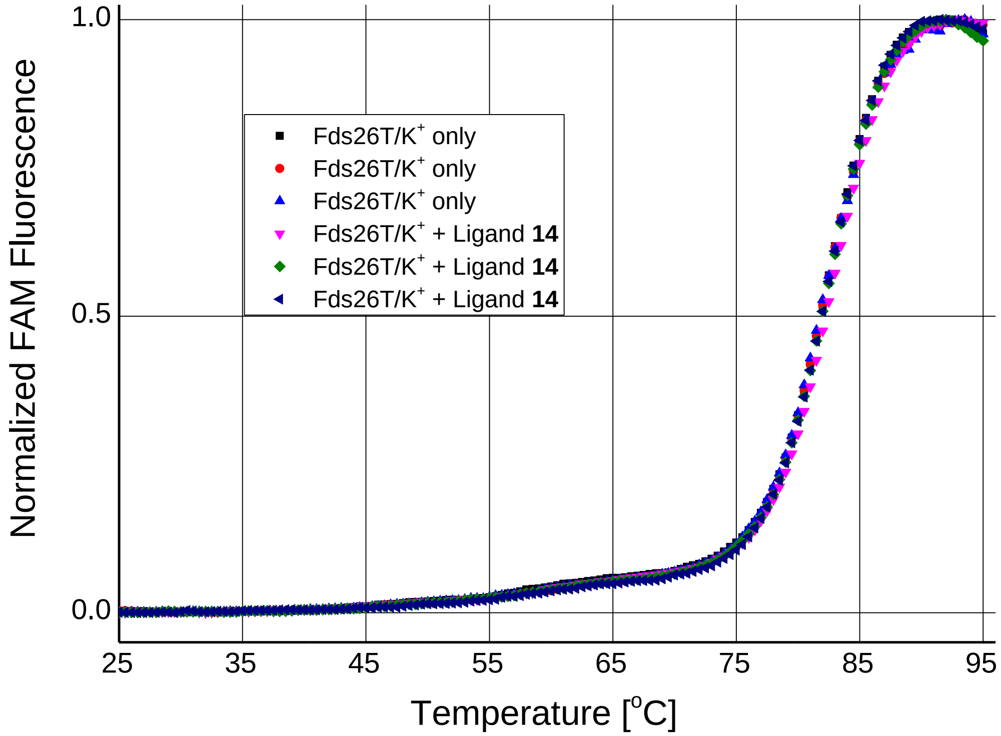

Fds26T/K^+/6mer (**13**):

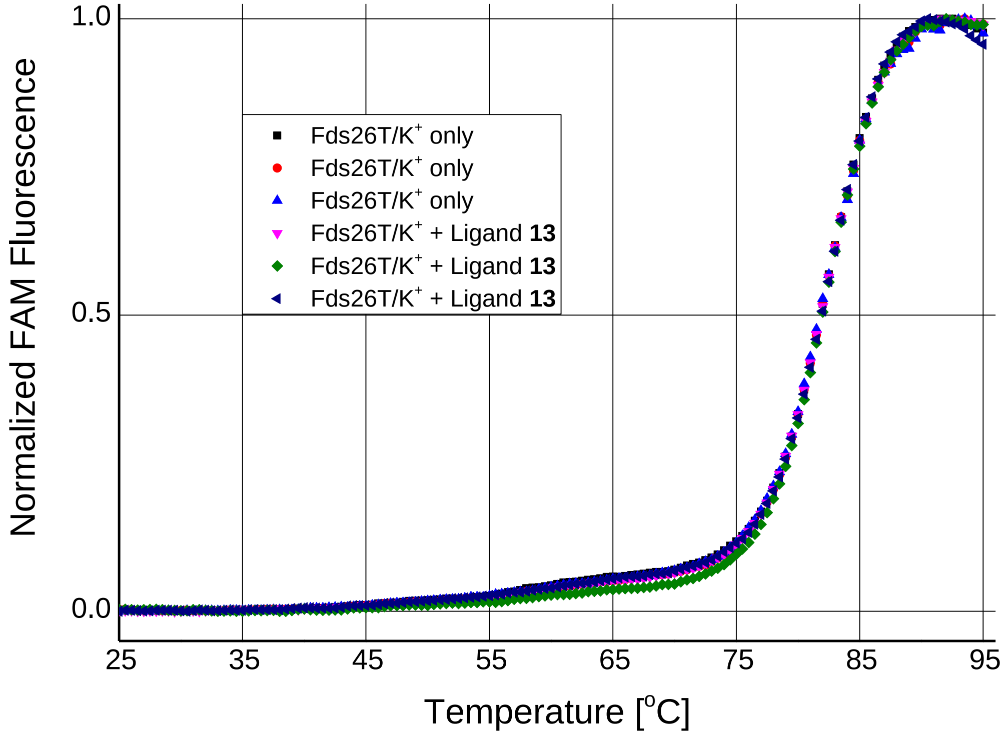

Fds26T/K^+/5mer (**10**):

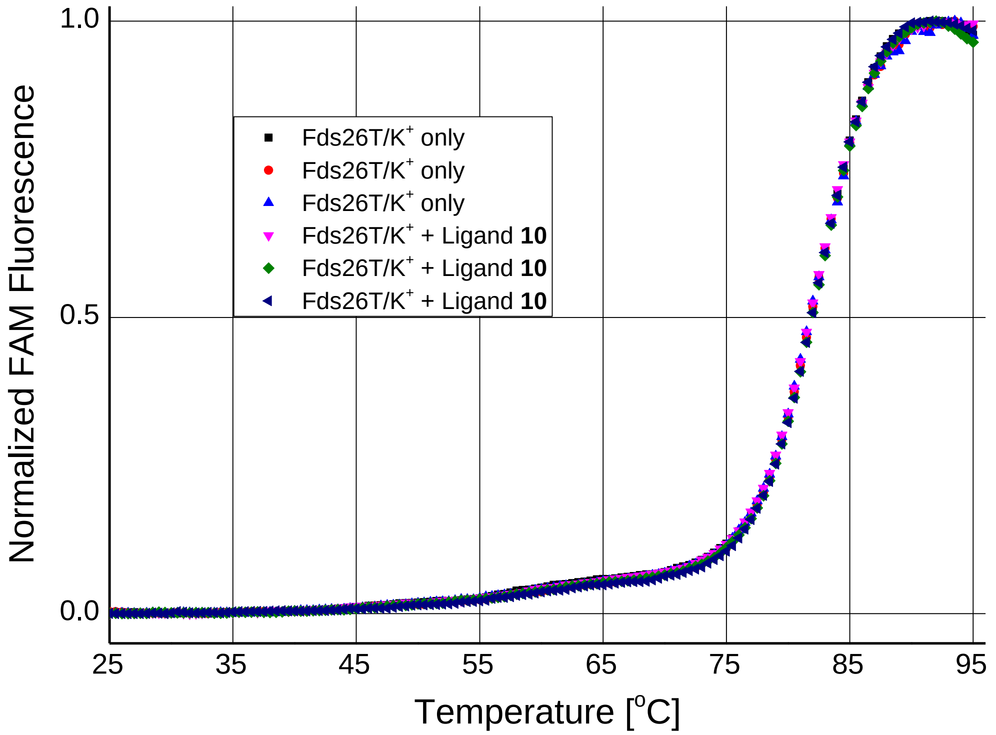

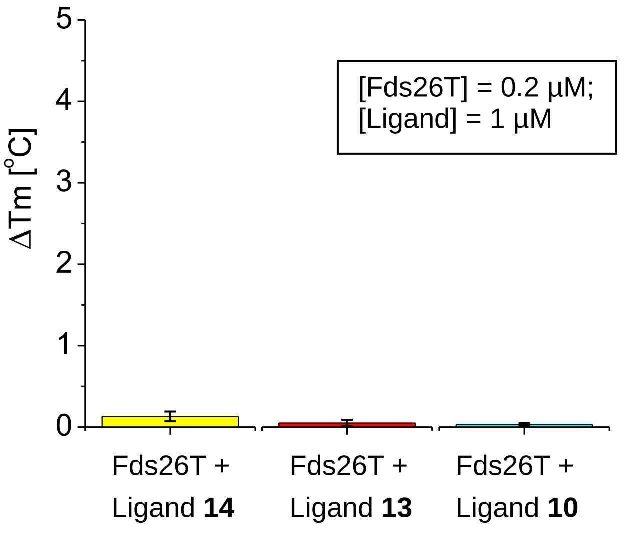

5
4
3
2
1
0
ΔTm [°C]
[Fds26T] = 0.2 µM;
[Ligand] = 1 µM
Fds26T + Ligand 14
Fds26T + Ligand 13
Fds26T + Ligand 10
